# Benefit, Harm, and Cost-effectiveness Associated With Magnetic Resonance Imaging Before Biopsy in Age-based and Risk-stratified Screening for Prostate Cancer

**DOI:** 10.1001/jamanetworkopen.2020.37657

**Published:** 2021-03-11

**Authors:** Thomas Callender, Mark Emberton, Stephen Morris, Paul D. P. Pharoah, Nora Pashayan

**Affiliations:** 1 Department of Applied Health Research, University College London, London, United Kingdom; 2 Division of Surgery and Interventional Science, University College London, London, United Kingdom; 3 Department of Public Health and Primary Care, University of Cambridge, Cambridge, United Kingdom; 4 Department of Oncology, University of Cambridge, Cambridge, United Kingdom

## Abstract

**Importance:**

If magnetic resonance imaging (MRI) mitigates overdiagnosis of prostate cancer while improving the detection of clinically significant cases, including MRI in a screening program for prostate cancer could be considered.

**Objective:**

To evaluate the benefit-harm profiles and cost-effectiveness associated with MRI before biopsy compared with biopsy-first screening for prostate cancer using age-based and risk-stratified screening strategies.

**Design, Setting, and Participants:**

This decision analytical model used a life-table approach and was conducted between December 2019 and July 2020. A hypothetical cohort of 4.48 million men in England aged 55 to 69 years were analyzed and followed-up to 90 years of age.

**Exposures:**

No screening, age-based screening, and risk-stratified screening in the hypothetical cohort. Age-based screening consisted of screening every 4 years with prostate-specific antigen between the ages of 55 and 69 years. Risk-stratified screening used age and polygenic risk profiles.

**Main Outcomes and Measures:**

The benefit-harm profile (deaths from prostate cancer, quality-adjusted life-years, overdiagnosis, and biopsies) and cost-effectiveness (net monetary benefit, from a health care system perspective) were analyzed. Both age-based and risk-stratified screening were evaluated using a biopsy-first and an MRI-first diagnostic pathway. Results were derived from probabilistic analyses and were discounted at 3.5% per annum.

**Results:**

The hypothetical cohort included 4.48 million men in England, ranging in age from 55 to 69 years (median, 62 years). Compared with biopsy-first age-based screening, MRI-first age-based screening was associated with 0.9% (1368; 95% uncertainty interval [UI], 1370-1409) fewer deaths from prostate cancer, 14.9% (12 370; 95% UI, 11 100-13 670) fewer overdiagnoses, and 33.8% (650 500; 95% UI, 463 200-907 000) fewer biopsies. At 10-year absolute risk thresholds of 2% and 10%, MRI-first risk-stratified screening was associated with between 10.4% (7335; 95% UI, 6630-8098) and 72.6% (51 250; 95% UI, 46 070-56 890) fewer overdiagnosed cancers, respectively, and between 21.7% fewer MRIs (412 100; 95% UI, 411 400-412 900) and 53.5% fewer biopsies (1 016 000; 95% UI, 1 010 000-1 022 000), respectively, compared with MRI-first age-based screening. The most cost-effective strategies at willingness-to-pay thresholds of £20 000 (US $26 000) and £30 000 (US $39 000) per quality-adjusted life-year gained were MRI-first risk-stratified screening at 10-year absolute risk thresholds of 8.5% and 7.5%, respectively.

**Conclusions and Relevance:**

In this decision analytical model of a hypothetical cohort, an MRI-first diagnostic pathway was associated with an improvement in the benefit-harm profile and cost-effectiveness of screening for prostate cancer compared with biopsy-first screening. These improvements were greater when using risk-stratified screening based on age and polygenic risk profile and may warrant prospective evaluation.

## Key Points

**Question:**

Is magnetic resonance imaging (MRI) before biopsy associated with improved benefit-harm and cost-effectiveness profiles compared with biopsy-first screening for prostate cancer using risk-stratified and age-based strategies?

**Findings:**

In this decision analytical model of a hypothetical cohort of 4.48 million men aged 55 to 69 years, MRI-first screening strategies were associated with a more favorable benefit-harm profile and with improved cost-effectiveness compared with biopsy-first strategies. The MRI-first risk-stratified screening strategies were more cost-effective than MRI-first age-based screening and were associated with less overdiagnosis and a comparable number of prevented deaths from prostate cancer.

**Meaning:**

Risk-stratified screening using MRI before biopsy was associated with improvements in the benefit-harm profile and cost-effectiveness compared with biopsy-first screening for prostate cancer, suggesting that this strategy should be evaluated prospectively.

## Teaser

This decision analytical model uses a hypothetical cohort of men in England to evaluate whether magnetic resonance imaging before biopsy for prostate cancer screening is associated with improvements in benefit-harm and cost-effectiveness profiles compared with biopsy-first screening.

## Introduction

The use of multiparametric magnetic resonance imaging (MRI) as a triage test before biopsy in men with a clinical suspicion of prostate cancer has been shown both to be cost-effective^1^ and to be associated with a number of benefits, including the avoidance of unnecessary biopsies in approximately one-third of men, an improved detection rate of clinically significant cancer, and a reduction in the detection of clinically insignificant cancer.^2,3,4,5^ Although screening using prostate-specific antigen (PSA) is associated with a 20% reduction in prostate cancer–specific mortality,^6^ the harms of overdiagnosis and overtreatment are considered to outweigh this benefit in most men.^7^ As a result, formal, population-based screening is not currently recommended in any jurisdiction. Overdiagnosed cancers are those that in the absence of screening would neither be detected nor impact individuals during their lifetime.^8^ Offering MRI before biopsy in a population-based screening program for prostate cancer would entail additional cost. However, this cost may be offset by fewer biopsies and a reduction in the number of men diagnosed with prostate cancer, largely by mitigating overdiagnosis.

A previous modeling study^9^ showed that a risk-stratified screening program based on age and polygenic profile may be more cost-effective and preserve the mortality benefits associated with age-based screening with PSA while reducing the number of cancers overdiagnosed. However, the benefit-harm profile and cost-effectiveness associated with a biopsy-first age-based screening program compared with those associated with biopsy-first risk-stratified screening and whether there are further gains associated with risk-stratified screening coupled with an MRI-first diagnostic pathway are unknown. An assessment of the outcomes associated with MRI using different screening strategies is necessary before designing a prospective evaluation of a prostate cancer screening program. In this decision analytical model, we evaluated MRI as a triage test before biopsy with age-based and polygenic risk–stratified screening strategies and assessed screening strategies associated with the greatest improvements in benefit-harm profiles and cost-effectiveness.

## Methods

### Model Structure

This decision analytical model, conducted between December 2019 and July 2020, used a life-table approach adapted from a model of polygenic risk–stratified screening for prostate cancer.^9^ This Markov model simulated a hypothetical cohort of men in no screening, age-based screening, and polygenic risk–stratified screening scenarios. The hypothetical cohort consisted of 4.48 million men aged 55 to 69 years, the mean population of men between these ages in England from 2013 to 2016, followed up to 90 years of age.^9,10^ The University College London Research Ethics Committee would deem this study exempt from ethical review and informed patient consent because it used only openly available data sources. The study followed the Consolidated Health Economic Evaluation Reporting Standards (CHEERS) reporting guideline.

In the hypothetical age-based screening cohorts, men received PSA testing every 4 years between the ages of 55 and 69 years in accordance with the European Randomized Study of Screening for Prostate Cancer (ERSPC).^11^ We used age and polygenic profiles to estimate the 10-year absolute risk of developing prostate cancer in the risk-stratified screening cohort. We varied the 10-year absolute risk thresholds at which individuals were eligible for screening from 2% to 10%. The age-specific proportion of men eligible for screening varies by risk; as an example, at a 2% absolute risk threshold, 49% of men would start screening at 55 years of age, with gradually more men being screened by 69 years of age. Only men above the 10-year absolute risk threshold started quadrennial screening with PSA at the age that they reached this threshold, with all screening ending at 69 years of age.

### Modeling MRI

In the screened cohorts, men were suspected to have prostate cancer if they had a PSA level ≥3 ng/mL, as per the core analyses of the ERSPC.^11^ To assess the consequences of MRI for screening, we modeled 2 diagnostic pathways: biopsy first and MRI first. In the modeled biopsy-first screening pathway, men with suspected prostate cancer (PSA level ≥3 ng/mL) next received a diagnostic biopsy. In the modeled MRI-first pathway, all men with a PSA level ≥3 ng/mL were offered an MRI. Those with abnormal MRI findings, defined as a Prostate Imaging–Reporting and Data System^12^ score ≥3, were subsequently offered a biopsy.

In the MRI-first cohorts, we adjusted incidence, mortality, and the cancer stage at diagnosis to reflect the detectability of clinically significant and insignificant cancers by MRI before biopsy (eAppendix in the Supplement). In the risk-stratified screening cohorts, we multiplied incidence and mortality by the age-specific relative risk of developing cancer in the higher and lower risk groups for each absolute risk threshold. We calculated overdiagnosed cancers by multiplying incident screening-detected cancers by the age-specific proportion estimated to be overdiagnosed,^13^ adjusting in the MRI-first cohorts for the reduction in clinically insignificant cancers detected by MRI.

### Polygenic Risk

The risk of receiving a diagnosis of prostate cancer varies among men. When combined, the 175 susceptibility loci for prostate cancer that have been identified in genome-wide association studies^14^ define a log-normal relative risk distribution with a variance of 0.68 (further details are given in the eAppendix in the Supplement).^9^ We used this distribution to evaluate the age-specific proportion of men eligible for screening by risk threshold as well as the proportion of total cancers expected to occur in these men. From this, we derived the age-specific relative risk of developing cancer among those above and below the threshold.

### Model Parameters and Outputs

Model parameters are shown in Table 1.^2,8,11,15,16,17,18,19,20,21,22,23,24,25,26^ Their underlying assumptions have been described previously^9^ and are available in the eAppendix in the Supplement. We derived estimates of the detectability of MRI for clinically significant and insignificant cancers from the systematic review and meta-analysis of Drost and colleagues^2^ and of misclassification using data from the Trio study.^16^ In this context, misclassification occurs when a cancer is reported as clinically insignificant on MRI rather than clinically significant. We generated a life table of prostate cancer incidence and mortality as well as mortality from other causes based on mean data from 2013 to 2016 from the Office for National Statistics.^10,27,28^

**Table 1.** Parameters of the Decision Analytical Model

Parameter	Central estimate (95% CI)	Parameterization in probabilistic analyses^a^	Source
Life table			
Relative reduction in prostate cancer–specific mortality with screening	0.80 (0.72-0.89)	SE = 0.06	^11^
Relative incidence of prostate cancer with screening	1.23 (1.03-1.48)	SE = 0.18	^15^
Proportion of overdiagnosed cancers	−0.62 + (Age ×0.014)	SE = 0.001	^8^
Relative reduction in advanced cancer at diagnosis if screened	0.85 (0.72-0.99)	SE = 0.07	^15^
Diagnostic pathway			
Detected using MRI before biopsy			
Reduction in clinically insignificant cancers	0.92 (0.90-0.94)	SE = 0.01	^2^
Increase in clinically significant cancers	1.02 (1.01-1.05)	SE = 0.01	^2^
Cancers misclassified by MRI as insignificant, %	2.76 (2.06-3.46)	SE = 0.004	^16^
Utility values			
General population utility	0.86 (0.85-0.88)	0.83 + [Gamma (4, 0.06) × 0.167]	^17^
Relative reduction in utility for those with prostate cancer	0.93 (0.88-1.00)^b^	0.88 + [Gamma (5, 0.05) × 0.20]	^18^
Costs, GBP in 2020 prices^c^			
Prostate-specific antigen testing	13 (9-18)	α = 33.9; β = 0.4	^19,20^
Polygenic risk stratification	25 (17-33)	α = 33.9; β = 0.7	^d^
Multiparametric MRI	379 (254-504)	α = 33.9; β = 11.2	^21^
Biopsy	581 (389-772)	α = 33.9; β = 17.1	^19,22,23^
Staging of diagnosed cancer	545 (365-725)	α = 33.9; β = 16.1	^19,22,23^
Active surveillance	5052 (3385-6719)^e^	α = 33.9; β = 149.1	^19,23,24^
Radical prostatectomy	9808 (6571-13 044)	α = 33.9; β = 289.5	^19,22,23^
Radical radiotherapy	6462 (4330-8594)	α = 33.9; β = 190.7	^19,22,23^
Brachytherapy	1832 (1228-2437)	α = 33.9; β = 54.1	^19,22,23^
Chemotherapy	8911 (5971-11 852)	α = 33.9; β = 263.0	^19,22,25^
Androgen-deprivation therapy	671 (449-892)	α = 33.9; β = 19.8	^9,22^
Palliation and death from prostate cancer	8204 (642-24 308)^f^	α = 1.8; β = 4625.9	^26^

Abbreviations: GBP, British pounds; MRI, magnetic resonance imaging.

^a^ The following distributions were used: log-normal for relative reductions and increases, β for proportions, and gamma for utilities and cost. All α and β refer to shape and scale, respectively.

^b^ Range.

^c^ To convert GBP to US dollars, multiply by 1.36.

^d^ Cost of polygenic risk stratification was empirical from personal communication of tariffs applied in the English National Health Service.

^e^ The eAppendix in the Supplement and Callender et al^9^ give further details.

^f^ 95% credible interval.

Outputs were the number of prostate cancers, deaths from prostate cancer, overdiagnosed cancers, biopsies, MRIs, life-years, quality-adjusted life-years (QALYs), and costs. We modeled costs from the perspective of the National Health Service in 2020 prices and derived the cost of polygenic screening from an empirical estimate. We included the following cost components: screening tests, diagnosis and assessment, treatment, and end-of-life care. We used literature-based estimates of treatment to calculate prostate cancer utilities. We applied a discount of 3.5% to all future costs and benefits, reflecting National Institute for Health and Care Excellence guidance.^29^

### Cost-effectiveness

We used net monetary benefit (NMB) to compare the cost-effectiveness of different screening interventions, calculated by subtracting costs accrued from the QALYs generated by an intervention multiplied by the willingness-to-pay threshold. The willingness-to-pay threshold reflects the value that a health system deems appropriate to pay for 1 year at full health; we used willingness-to-pay thresholds of £20 000 (US $26 000) and £30 000 (US $39 000), the range considered by the National Institute for Health and Care Excellence.^29^ The screening strategy with the highest NMB for a given willingness to pay was considered the most cost-effective.

### Statistical Analysis

We accounted for parameter uncertainty by running each scenario 10 000 times, on each occasion drawing parameter estimates for all variables simultaneously from an underlying distribution (Table 1). We present the mean of these probabilistic analyses throughout unless otherwise stated. We generated 95% uncertainty intervals (UIs) by using the values at the 2.5th and 97.5th centiles of the sorted probabilistic results. To reflect parameter uncertainty in the presentation of the results in the text, we rounded values to 4 significant digits.

We conducted scenario analyses to evaluate the consequences of different assumptions concerning the associations between changes in clinically insignificant and significant cancers detected by MRI, the costs of polygenic testing and MRI, varying overdiagnosis by polygenic risk, and uptake of PSA and risk-stratified screening. We performed all statistical analyses using Python, version 3.7 (Python Software Foundation).

## Results

The decision analytical model included a hypothetical cohort of 4.48 million men in England, ranging in age from 55 to 69 years (median, 62 years). The age distribution of the cohort is shown in eFigure 1 in the Supplement.

### Comparison of MRI-First Age-based and Risk-stratified Screening Strategies With No Screening

**Figure 1.**
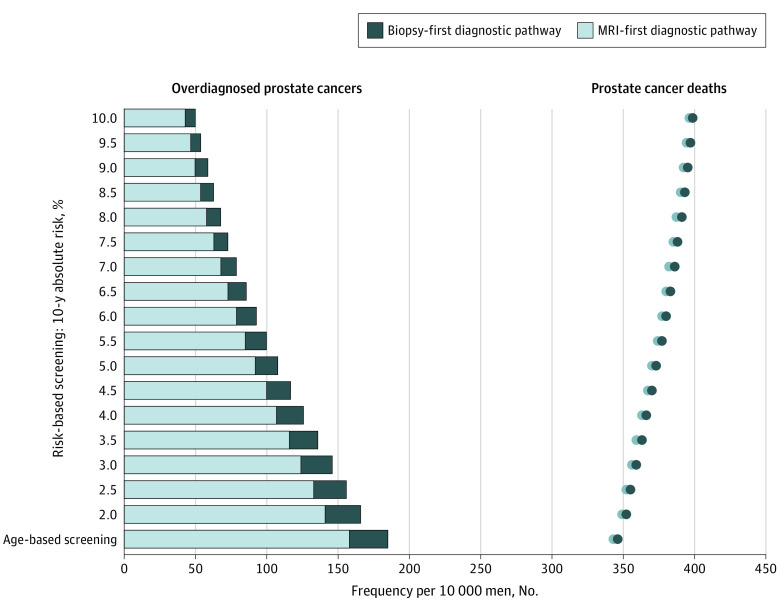
Overdiagnosed Cancers and Deaths From Prostate Cancer by Diagnostic Pathway MRI indicates magnetic resonance imaging.

Compared with no screening, MRI-first age-based screening was associated with 36 910 (95% CI, 33 720-40 040) fewer deaths from prostate cancer but 70 640 (95% UI, 63 100-79 070) overdiagnosed cancers, which was 1 in 4 screen-detected cancers (Figure 1). The MRI-first age-based screening strategy was associated with an increase of 994 000 (95% CI, 979 500-1 007 000) MRIs and 667 200 (95% UI, 662 100-669 400) additional biopsies.

As the risk threshold increased from 2% to 10%, MRI-first risk-stratified screening was associated with a decrease in the ratio of overdiagnosed cancers to prevented deaths from cancer from 1.8 to 1.5; minimizing this ratio improved the benefit-to-harm profile of screening. Compared with no screening at risk thresholds of 2% and 10%, MRI-first risk-stratified screening was associated with between 13 370 (95% CI, 12 640-14 070) and 34 450 (95% CI, 31 590-37 250) fewer deaths from prostate cancer and between 19 390 (95% CI, 17 030-22 180) and 63 300 (95% CI, 56 470-70 970) overdiagnoses. The MRI-first risk-stratified screening programs were associated with needing fewer additional resources because the proportion of men eligible for screening decreased as the risk threshold increased (Table 2). The relative risk of developing cancer compared with the mean risk among those eligible for screening increased as the risk threshold increased. As a result, compared with no screening, MRI-first risk-stratified screening was associated with a greater yield of cancers per MRI and biopsy and, consequently, with fewer scans and biopsies needed.

**Table 2.** Outcomes Associated With MRI-First Age-based Screening, MRI-First Risk-stratified Screening, and No Screening^a^

Screening strategy	No.								Costs, millions of GBP^d^	Cumulative eligible for screening, %^e^
	Cancer cases		Overdiagnosed cases	Deaths from prostate cancer	MRIs	Biopsies	Life-years	Quality-adjusted life-years		
	Prostate^b^	Screening detected^c^								
No screening	527 685	NA	NA	190 748	906 551	608 602	60 333 037	46 694 958	3471	0
Screening										
Age based	625 151	285 062	70 636	153 834	1 900 566	1 275 766	60 458 871	46 713 875	4382	100
Risk stratified, 10-y absolute risk, %										
2.0	605 904	254 286	63 302	156 299	1 488 417	999 127	60 449 805	46 716 499	4148	73
2.5	598 103	238 784	59 570	157 809	1 373 255	921 831	60 444 508	46 716 954	4046	64
3.0	590 515	222 804	55 704	159 449	1 277 309	857 432	60 438 787	46 717 174	3958	56
3.5	583 369	207 038	51 871	161 134	1 198 704	804 674	60 432 923	46 717 192	3884	49
4.0	576 768	191 885	48 172	162 809	1 134 780	761 769	60 427 103	46 717 046	3822	43
4.5	570 747	177 560	44 663	164 439	1 082 950	726 981	60 421 443	46 716 772	3770	38
5.0	565 301	164 160	41 368	166 004	1 040 961	698 799	60 416 013	46 716 399	3726	33
5.5	560 403	151 713	38 298	167 493	1 006 943	675 967	60 410 854	46 715 952	3690	29
6.0	556 017	140 204	35 452	168 899	979 374	657 464	60 405 983	46 715 449	3660	26
6.5	552 101	129 595	32 821	170 222	957 029	642 467	60 401 405	46 714 907	3635	23
7.0	548 612	119 834	30 394	171 463	938 923	630 316	60 397 116	46 714 339	3613	20
7.5	545 510	110 864	28 159	172 625	924 265	620 478	60 393 107	46 713 754	3596	18
8.0	542 754	102 625	26 102	173 710	912 419	612 527	60 389 365	46 713 160	3581	16
8.5	540 311	95 059	24 208	174 724	902 871	606 120	60 385 875	46 712 564	3569	15
9.0	538 146	88 111	22 467	175 670	895 208	600 977	60 382 622	46 711 970	3559	13
9.5	536 230	81 728	20 863	176 553	889 093	596 873	60 379 591	46 711 382	3551	12
10.0	534 536	75 862	19 388	177 378	884 250	593 624	60 376 768	46 710 804	3544	11

Abbreviations: GBP, British pounds; MRI, magnetic resonance imaging; NA, not applicable.

^a^ Outcomes among 4.48 million men in hypothetical cohorts followed up to age 90 years.

^b^ Prostate cancer cases in the risk-stratified program encompass those detected by screening in high-risk groups between ages 55 and 69 years, those clinically detected in high-risk groups after 69 years of age when screening stopped, and those clinically detected in low-risk groups.

^c^ Screening-detected cancers were cancers detected with screening between the ages of 55 and 69 years.

^d^ To convert GBP to US dollars, multiply by 1.36.

^e^ Cumulative proportion of the population above the risk threshold and therefore eligible for screening between the ages of 55 and 69 years (more details are given in eTable 1 in the Supplement). The no-screening scenario assumes that men with clinically suspected cancer will have an MRI before biopsy in accordance with 2019 National Institute for Health and Care Excellence guidelines.^29^

### Comparison of MRI-First and Biopsy-First Age-based Screening

In comparison with a biopsy-first diagnostic approach, MRI-first age-based screening was associated with 0.9% (1368; 95% UI, 1370-1409) fewer deaths from prostate cancer, 14.9% (12 370; 95% UI, 11 100-13 670) fewer overdiagnosed cancers, and 33.8% (650 500; 95% UI, 463 200-907 000) fewer biopsies. This translated into an associated increase of 0.03% (15 840; 95% UI, 11 170-25 850) total QALYs and 0.008% (4600; 95% UI, 4602-4772) total life-years (Figure 1, Table 2, and eTable 2 in the Supplement) and an associated decrease in the ratio of overdiagnosis to prevented deaths from prostate cancer from 2.2 to 1.9. The costs associated with MRI-first compared with biopsy-first age-based screening were lower despite an associated 4.8-fold increase in the number of MRIs (1.5 million; 95% UI, 1.47 million-1.53 million).

### Comparison of MRI-First and Biopsy-First Risk-stratified Screening

In comparison with biopsy-first risk-stratified screening, MRI-first risk-stratified screening was associated with fewer deaths from prostate cancer, fewer overdiagnosed cancers, just less than half the number of biopsies, and an increase in QALYs gained at a lower cost (Table 2 and eTable 2 in the Supplement). At a 10-year absolute risk threshold of 2%, MRI-first risk-stratified screening was associated with a 3.9-fold greater number of scans required (1 102 000; 95% UI, 1 074 000-1 136 000) compared with a biopsy-first risk-stratified screening program, decreasing to a 2.6-fold increase (545 900; 95% UI, 503 000-596 300) at a risk threshold of 10%.

### Comparison of MRI-First Age-based Screening With MRI-First Risk-stratified Screening

Compared with MRI-first age-based screening, MRI-first risk-stratified screening was associated with fewer harms (overdiagnoses and biopsies) and lower costs but with more deaths from prostate cancer. At 10-year absolute risk thresholds of 2% and 10%, MRI-first risk-stratified screening was associated with between 10.4% (7335; 95% UI, 6630-8098) and 72.6% (51 250; 95% UI, 46 070-56 890) fewer overdiagnosed cancers, respectively, and between 21.7% fewer MRIs (412 100; 95% UI, 411 400-412 900) and 53.5% fewer biopsies (1 016 000; 95% UI, 1 010 000-1 022 000), respectively, compared with MRI-first age-based screening (Table 2). In comparison with MRI-first age-based screening, MRI-first risk-stratified screening was associated with more QALYs at all risk thresholds below 7.5% and with progressively lower costs as the risk threshold increased from 2.0% to 10.0%. However, MRI-first risk-stratified screening was associated with between 1.6% (2465; 95% UI, 2133-2794) and 15.3% (23 540; 95% UI, 21 080-25 970) more deaths from prostate cancer at risk thresholds of 2.0% and 10.0%, respectively.

### Cost-effectiveness

**Figure 2.**
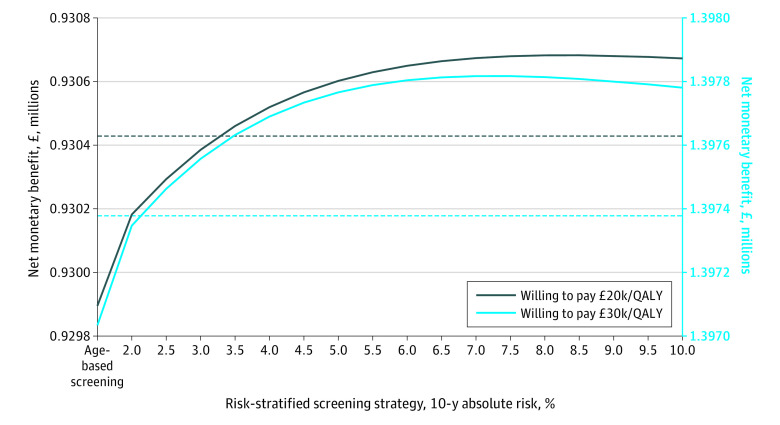
Net Monetary Benefit Associated With Age-based, Risk-stratified, and No-Screening Strategies Evaluated With a Magnetic Resonance Imaging–First Diagnostic Pathway The grey dashed line represents no screening at a willingness to pay of £20 000 (US $26 000); and the blue dashed line, at £30 000 (US $39 000). To convert British pounds to US dollars, multiply by 1.36. QALY indicates quality-adjusted life-year.

All MRI-first risk-stratified screening scenarios at thresholds of 3.5% or greater were associated with an NMB greater than that of no screening at a willingness-to-pay threshold of £20 000 (US $26 000) (Figure 2; cost-effectiveness acceptability curves are shown in eFigure 2 in the Supplement). MRI-first age-based screening was associated with the lowest NMB and was the least cost-effective MRI-first screening strategy at willingness-to-pay thresholds of both £20 000 (US $26 000) and £30 000 (US $39 000) per QALY gained. The strategies associated with the highest NMB at willingness-to-pay thresholds of £20 000 (US $26 000) and £30 000 (US $39 000) per QALY gained were MRI-first risk-stratified screening at a risk threshold of 8.5% and 7.5%, respectively (the cost-effectiveness acceptability frontier is presented in eFigure 3 in the Supplement, and the NMB of MRI-first and biopsy-first screening scenarios are given in eFigure 4 in the Supplement).

### Scenario Analyses

In the Prostate Evaluation for Clinically Important Disease: Sampling Using Image Guidance or Not? (PRECISION) trial,^30^ MRI before biopsy was associated with a 13% (95% CI, 7%-19%) reduction in clinically insignificant cancers detected and a 12% (95% CI, 4%-20%) increase in clinically significant cancers detected. Using these parameters, MRI-first screening strategies were associated with an improvement in the benefit-harm profile and NMB (eFigure 5 in the Supplement). However, age-based screening was associated with reduced cost-effectiveness compared with risk-stratified screening (eTable 5 and eFigure 6 in the Supplement). The cost-effectiveness of MRI-first screening strategies was insensitive to the cost of an MRI scan (baseline of £380 [US $494] to £100 [US $130]) (eFigure 7 in the Supplement). In contrast, MRI-first risk-stratified screening strategies were sensitive to the cost of risk stratification (varied from £25 [US $33] to £100 [US $130]) (eFigure 8 in the Supplement). A 75% uptake of PSA screening was associated with greater cost-effectiveness; however, MRI-first risk-stratified screening was insensitive to a 75% (baseline 100%) uptake of polygenic risk stratification (eFigure 9 in the Supplement). Overdiagnosis has been shown to vary inversely by polygenic risk^8^; in this scenario, the ratio of prevented deaths from prostate cancer to overdiagnosed cancers was associated with further improvement in risk-stratified screening (eFigure 10 in the Supplement).

## Discussion

This decision analytical model showed that an MRI-first diagnostic pathway was associated with an improved benefit-harm profile for prostate cancer screening compared with a biopsy-first diagnostic pathway. This improvement was associated with a reduction in biopsies, overdiagnoses, and deaths from prostate cancer. Moreover, an MRI-first approach was associated with more QALYs at reduced costs compared with a biopsy-first diagnostic pathway.

In addition, we showed that these benefits were greater when risk-stratified screening was combined with an MRI-first diagnostic pathway. By tailoring screening to men at higher absolute risk of developing prostate cancer, MRI-first risk-stratified screening was associated with preventing a number of deaths from prostate cancer comparable with the number of deaths prevented by MRI-first age-based screening. MRI-first risk-stratified screening was also associated with a 10.4% to 72.6% lower probability of overdiagnosis and 21.7% to 53.5% fewer unnecessary biopsies as well as an improvement in the cost-effectiveness of a screening program (Figure 1, Figure 2, and Table 2). Increasing the risk threshold was associated with a lower ratio of overdiagnosed cancers to deaths from prostate cancer. Eligibility for screening became more strict as the risk threshold increased, such that there were fewer screening-detected and potentially overdiagnosed cancers.

Of all strategies studied, MRI-first risk-stratified screening at a 10-year absolute risk threshold of 3.5% was associated with the greatest number of QALYs gained, beyond which the QALYs gained diminished (Table 2 and eTable 2 in the Supplement). As the risk threshold increased, risk-stratified screening was associated with a greater decrease in costs compared with QALYs generated, such that the NMB associated with MRI-first risk-stratified screening began to plateau at a risk threshold of approximately 7% to 8%. This reflects an association with a lower proportion of men expected to be overdiagnosed and with a decrease in the number of men who would be eligible for screening as the risk threshold increased. The benefits associated with screening (mortality reduction and QALYs gained) decreased as the risk threshold increased because the proportion of men eligible for screening became increasingly smaller (Table 2). As a result, the ideal risk threshold for screening would represent a balance between that which minimizes overdiagnosis and maximizes mortality reduction and QALYs gained at an acceptable cost-effectiveness ratio.^31^

There are several ways that a risk-stratified screening program might be further tailored. The screening interval could be varied by risk if the sojourn time—the time that a cancer remains in a detectable but preclinical state—differs by risk level. This strategy might reduce the number of interval cancers and improve the mortality reduction of the program. To our knowledge, there currently are no data on how the sojourn time varies with risk level; thus, this should be the subject of future work. Alternative strategies also include varying the starting and stopping ages for screening by risk level. Different prognostic markers, such as the 4-kallikrein score as a triage test before biopsy, also warrant comparative analyses.^32^

### Strengths and Limitations

This study has strengths. To our knowledge, there are no comparable models of prostate cancer incorporating MRI and no trials of MRI-first screening. Using a life-table approach, we based the model on well-validated population data, allowing us to calibrate the model (eFigures 11 and 12 in the Supplement), minimize assumptions, and maximize model clarity while using probabilistic analyses to account for parameter uncertainty. We used meta-analyses as the basis of the inputs when possible, accounted for misclassification of cancers by MRI, and ran sensitivity analyses to explore alternative scenarios. Rather than making assumptions regarding the association between polygenic risk and indolent and nonindolent cancers, we used age-specific probabilities of overdiagnosis for transparency. To reflect a countrywide screening program involving radiological centers with varying degrees of experience with MRI, we used conservative baseline estimates of the detection level of MRI for clinically significant and insignificant cancers. Centers with substantial experience with MRI have shown greater reductions in detection of clinically insignificant cancers and greater increases in detection of clinically significant cancers.^5^ In addition, we used NMB to facilitate the comparison of multiple alternatives and to avoid assumptions of which pairwise comparisons are most appropriate.^33^

This study also has limitations. We extrapolated the detection rate of MRI for clinically suspected cancer to a screened hypothetical cohort. Magnetic resonance imaging has been shown to distinguish between clinically significant and insignificant cancers.^2,3^ However, the proportion of cancers deemed clinically insignificant that will progress to become clinically significant and the implications of an MRI-first diagnostic pathway for long-term prostate cancer outcomes remain unknown. In addition, risk-stratified screening may be associated with greater reductions in overdiagnosis and mortality than was found in our study. In the absence of screening data, we assumed in base-case analyses that overdiagnosis and mortality would not differ from that reported in age-based screening trials. The base-case model may therefore underestimate the reduction in overdiagnosis (sensitivity analyses are provided in eFigure 9 in the Supplement), and the assumption that risk-stratified screening would not be associated with a lower relative risk of death from prostate cancer among those screened may not hold.^34^

## Conclusions

In this decision analytical model of a hypothetical cohort of men, an MRI-first diagnostic pathway was associated with an improved benefit-harm profile and cost-effectiveness of screening for prostate cancer. The improvement associated with an MRI-first pathway was greater with risk-stratified screening based on age and polygenic risk profile. Prospective evaluation of an MRI-first risk-stratified screening program, including implementation research, appears to be needed.

## Notes

Supplement. **eAppendix.** **eTable 1.** Age-specific cumulative proportion eligible for screening by 10-year absolute risk threshold **eTable 2.** Outcomes of biopsy-first no screening and biopsy-first screening for prostate cancer **eTable 3.** Outcomes of biopsy-first no screening and biopsy-first screening for prostate cancer per 10,000 men **eTable 4.** Outcomes of MRI-first no screening and MRI-first screening for prostate cancer per 10,000 men **eFigure 1. **Population age distribution **eFigure 2.** Cost-effectiveness acceptability curves showing MRI-first screening strategies **eFigure 3.** Cost-effectiveness acceptability frontier showing MRI-first screening strategies **eFigure 4.** Net monetary benefit of MRI-first and biopsy-first strategies **eTable 5.** Outcomes of no screening and MRI-first screening for prostate cancer per 10,000 men under assumptions from the PRECISION trial **eFigure 5.** Benefit/harm profile per 10,000 men with MRI-first screening using parameter estimates from the PRECISION trial **eFigure 6.** Net monetary benefit per 10,000 men of MRI-first screening and no screening using parameter estimates from the PRECISION trial **eFigure 7.** Net monetary benefit per 10,000 men of MRI-first screening and no screening at different costs of MRI **eFigure 8.** Net monetary benefit per 10,000 men of MRI-first screening and no screening at different costs of polygenic risk stratification **eFigure 9.** Net monetary benefit per 10,000 men of MRI-first screening and no screening at 75% uptake of PSA screening and polygenic risk stratification **eFigure 10.** Overdiagnosed cancers and prostate cancer deaths prevented per 10,000 men of MRI-first risk-stratified compared to MRI-first age-based screening when overdiagnosis varies by polygenic risk **eFigure 11.** Observed vs predicted incidence rate **eFigure 12.** Observed vs predicted mortality rate **eReferences**
