# Supplementary material for: Benefit, Harm, and Cost-effectiveness Associated With Magnetic Resonance Imaging Before Biopsy in Age-based and Risk-stratified Screening for Prostate Cancer
Source: JAMA Netw Open. 2021 Mar 11;4(3):e2037657. doi: 10.1001/jamanetworkopen.2020.37657 (PMC7953309; doi:10.1001/jamanetworkopen.2020.37657)
Supplement: Supplement. — eAppendix. eTable 1. Age-specific cumulative proportion eligible for screening by 10-year absolute risk threshold eTable 2. Outcomes of biopsy-first no screening and biopsy-first screening for prostate cancer eTable 3. Outcomes of biopsy-first no screening and biopsy-first screening for prostate cancer per 10,000 men eTable 4. Outcomes of MRI-first no screening and MRI-first screening for prostate cancer per 10,000 men eFigure 1. Population age distribution eFigure 2. Cost-effectiveness acceptability curves showing MRI-first screening strategies eFigure 3. Cost-effectiveness acceptability frontier showing MRI-first screening strategies eFigure 4. Net monetary benefit of MRI-first and biopsy-first strategies eTable 5. Outcomes of no screening and MRI-first screening for prostate cancer per 10,000 men under assumptions from the PRECISION trial eFigure 5. Benefit/harm profile per 10,000 men with MRI-first screening using parameter estimates from the PRECISION trial eFigure 6. Net monetary benefit per 10,000 men of MRI-first screening and no screening using parameter estimates from the PRECISION trial eFigure 7. Net monetary benefit per 10,000 men of MRI-first screening and no screening at different costs of MRI eFigure 8. Net monetary benefit per 10,000 men of MRI-first screening and no screening at different costs of polygenic risk stratification eFigure 9. Net monetary benefit per 10,000 men of MRI-first screening and no screening at 75% uptake of PSA screening and polygenic risk stratification eFigure 10. Overdiagnosed cancers and prostate cancer deaths prevented per 10,000 men of MRI-first risk-stratified compared to MRI-first age-based screening when overdiagnosis varies by polygenic risk eFigure 11. Observed vs predicted incidence rate eFigure 12. Observed vs predicted mortality rate eReferences [file jamanetwopen-e2037657-s001.pdf]

Supplementary Online Content

Callender T, Emberton M, Morris S, Pharoah PDP, Pashayan N. Benefit, harm, and cost-effectiveness associated with magnetic resonance imaging before biopsy in age-based and risk-stratified screening for prostate cancer. *JAMA Netw Open*. 2021;4(3):e2037657. doi:10.1001/jamanetworkopen.2020.37657

eAppendix.
eTable 1. Age-specific cumulative proportion eligible for screening by 10-year absolute risk threshold
eTable 2. Outcomes of biopsy-first no screening and biopsy-first screening for prostate cancer
eTable 3. Outcomes of biopsy-first no screening and biopsy-first screening for prostate cancer per 10,000 men
eTable 4. Outcomes of MRI-first no screening and MRI-first screening for prostate cancer per 10,000 men
eFigure 1. Population age distribution
eFigure 2. Cost-effectiveness acceptability curves showing MRI-first screening strategies
eFigure 3. Cost-effectiveness acceptability frontier showing MRI-first screening strategies
eFigure 4. Net monetary benefit of MRI-first and biopsy-first strategies
eTable 5. Outcomes of no screening and MRI-first screening for prostate cancer per 10,000 men under assumptions from the PRECISION trial
eFigure 5. Benefit/harm profile per 10,000 men with MRI-first screening using parameter estimates from the PRECISION trial
eFigure 6. Net monetary benefit per 10,000 men of MRI-first screening and no screening using parameter estimates from the PRECISION trial
eFigure 7. Net monetary benefit per 10,000 men of MRI-first screening and no screening at different costs of MRI
eFigure 8. Net monetary benefit per 10,000 men of MRI-first screening and no screening at different costs of polygenic risk stratification
eFigure 9. Net monetary benefit per 10,000 men of MRI-first screening and no screening at 75% uptake of PSA screening and polygenic risk stratification
eFigure 10. Overdiagnosed cancers and prostate cancer deaths prevented per 10,000 men of MRI-first risk-stratified compared to MRI-first age-based screening when overdiagnosis varies by polygenic risk
eFigure 11. Observed vs predicted incidence rate
eFigure 12. Observed vs predicted mortality rate
eReferences

This supplementary material has been provided by the authors to give readers additional information about their work.

eAppendix

Polygenic risk

In England, the 10-year absolute risk of developing prostate cancer rises from 1.3% at age 50 to a peak of 7.2% at the age of 71.[1] Common genetic susceptibility variants explain between 52% and 63% of the population variation in prostate cancer risk, making it the most heritable form of cancer.[2,3] Common susceptibility loci are assumed to interact log-additively, leading to a lognormal distribution of polygenic risk in the population on a relative risk scale.[4] Known prostate cancer susceptibility variants define a log relative risk distribution of 0.68.[5] From this we derived the age-specific proportion of men above each 10-year absolute risk threshold, and the proportion of all cancers that would be diagnosed in these men.[1] These proportions were used to calculate the age-specific relative risk of developing prostate cancer in those men above and below the 10-year absolute risk thresholds.

Men in the highest 10% of this risk distribution have a risk of developing prostate cancer of ~2.5 relative to the mean whilst those in the lowest 10% of the distribution have a relative risk of approximately 0.2.

The cumulative age-specific proportions receiving at least one screen at 10-year absolute risk thresholds varying from 2% to 10% are shown in eTable 1.

eTable 1: Age-specific cumulative proportion eligible for screening by 10-year absolute risk threshold

	Age														
**10-year absolute risk threshold**	**55**	**56**	**57**	**58**	**59**	**60**	**61**	**62**	**63**	**64**	**65**	**66**	**67**	**68**	**69**
2.0%	0.46	0.50	0.52	0.55	0.58	0.60	0.62	0.64	0.65	0.67	0.68	0.70	0.71	0.72	0.73
2.5%	0.36	0.39	0.42	0.44	0.47	0.49	0.51	0.54	0.55	0.57	0.59	0.60	0.62	0.63	0.64
3.0%	0.28	0.31	0.33	0.36	0.38	0.41	0.43	0.45	0.47	0.49	0.50	0.52	0.54	0.55	0.56
3.5%	0.22	0.25	0.27	0.29	0.31	0.34	0.36	0.38	0.40	0.42	0.43	0.45	0.47	0.48	0.49
4.0%	0.17	0.20	0.22	0.24	0.26	0.28	0.30	0.32	0.34	0.36	0.37	0.39	0.41	0.42	0.43
4.5%	0.14	0.16	0.18	0.20	0.21	0.23	0.25	0.27	0.29	0.31	0.32	0.34	0.35	0.37	0.38
5.0%	0.11	0.13	0.14	0.16	0.18	0.20	0.21	0.23	0.25	0.26	0.28	0.30	0.31	0.32	0.33
5.5%	0.09	0.11	0.12	0.13	0.15	0.17	0.18	0.20	0.21	0.23	0.24	0.26	0.27	0.28	0.29
6.0%	0.07	0.09	0.10	0.11	0.13	0.14	0.16	0.17	0.18	0.20	0.21	0.23	0.24	0.25	0.26
6.5%	0.06	0.07	0.08	0.09	0.11	0.12	0.13	0.15	0.16	0.17	0.19	0.20	0.21	0.22	0.23
7.0%	0.05	0.06	0.07	0.08	0.09	0.10	0.12	0.13	0.14	0.15	0.16	0.17	0.19	0.20	0.20
7.5%	0.04	0.05	0.06	0.07	0.08	0.09	0.10	0.11	0.12	0.13	0.14	0.15	0.16	0.17	0.18
8.0%	0.04	0.04	0.05	0.06	0.07	0.08	0.09	0.10	0.11	0.12	0.13	0.14	0.15	0.16	0.16
8.5%	0.03	0.04	0.04	0.05	0.06	0.07	0.08	0.08	0.09	0.10	0.11	0.12	0.13	0.14	0.15
9.0%	0.03	0.03	0.04	0.04	0.05	0.06	0.07	0.07	0.08	0.09	0.10	0.11	0.12	0.12	0.13
9.5%	0.02	0.03	0.03	0.04	0.04	0.05	0.06	0.07	0.07	0.08	0.09	0.10	0.10	0.11	0.12
10%	0.02	0.02	0.03	0.03	0.04	0.04	0.05	0.06	0.06	0.07	0.08	0.09	0.09	0.10	0.11

The cumulative proportion of the population eligible for a screen by age and 10-year absolute risk threshold. For example, at a risk threshold of 2%, at the age of 55 46.4% of the population are eligible for screening. At 56, 53.4% of individuals are eligible for screening. There are 341,434 men aged 55 and 330,382 aged 56. Therefore, the cumulative proportion screened by age 56 is (341,434*0.464 + 330,382*0.534) / (341,434+330,382) = 0.5. The age-specific population and the proportion eligible for screening are available with the source code for this analysis https://github.com/callta/PRIMARI.

Costing descriptions

A detailed description of the costs used, and their derivation, has been published elsewhere.[1] We have adjusted the cost of cancer staging and assessment, and that of active surveillance to reflect the updated 2019 National Institute for Health and Care Excellence (NICE) guidelines.[6]

Prostate biopsy costs were based on NICE estimates of the cost of a TRUS and perineal biopsy with relevant histopathology (£311.79 and £652.40, respectively).[7] The 2018 National Prostate Cancer Audit showed that 88% of men with prostate cancer in England had a TRUS biopsy and 12% a perineal biopsy. An assumed 1.4% will be admitted to hospital post-biopsy with complications,[8] with each hospital admission for sepsis costing an average of £1859 (WJ06G/H/J).[9] The final cost for a biopsy was £378.68 ((352.66 + (0.014 x 1859)).

The last year for which NHS Reference Costs were published was 2015/2016. We have therefore used the hospital services index of the Consumer Price Index (CPI) as published by the UK Office for National Statistics to inflate the values from 2015 to January 2020, over which period there has been a 20% rise in prices.[10]

Assessing suspected prostate cancer – if MRI suggestive of prostate cancer			
**Stage of pathway**	**Description**	**Items**	**Cost (£)**
Diagnosis & Staging	Biopsy	1	378.68
	Urological appointment	1	105.19
Total cost per patient (including inflation of 20%)			580.66

We assumed that those who have a positive biopsy would not require a further MRI.

Assessing suspected prostate cancer – if biopsy positive			
**Stage of pathway**	**Description**	**Items**	**Cost (£)**
Diagnosis & Staging	Isotope bone scan	1	242
	Urological MDT	1	107
	Urological appointment	1	105.19
Total cost per patient (including inflation of 20%)			545.03

Following the ProtecT trial,[11] we assumed that 45% of those eligible will have active surveillance for a period of 10-years, with the remaining 55% having either radical prostatectomy or radiotherapy.[1]

Active surveillance – 2019 NICE protocol			
**Year(s)**	**Description**	**Items**	**Cost (£)**
1	PSA test	3	33.78
	Urological appointment (digital rectal examination [DRE])	2	210.38
2-4	PSA test	2	67.56
	Urological appointment (DRE)	2	631.14
5 onwards	PSA test	2 per year for 5 years	112.60
	Urological appointment (DRE)	1 per year for 5 years	525.95
Total cost per patient (including inflation of 20%)			1897.69
Note, the total cost of active surveillance used in the model assumes 45% will have active surveillance over 10 years whilst 55% will have radical therapy. Active surveillance involves an MRI in those who have not had one already. The number and cost of MRI is accounted for elsewhere in the model.			

Resource use

Magnetic Resonance Imaging

In the screened cohorts, we estimated the number of multi-parametric Magnetic Resonance Imaging (MRI) scans by multiplying the number of individuals screened by the age-specific proportion estimated to have a PSA of ≥3ng/ml, derived from the ProtecT trial.[12]

Biopsies

In the non-screened cohort, the number of biopsies was estimated from PROMIS, in which there were 1.88 biopsies for every cancer detected in a clinically-suspected cohort diagnosed using MRI prior to biopsy.[13] This was modelled in probabilistic analyses using a normal distribution and a standard deviation of 0.1.

Two recent meta-analyses have shown that MRI prior to biopsy leads to a third of biopsies being avoided amongst cohorts with clinically-suspected prostate cancer.[14,15] We assume that under real-world scenarios 20% of those with an MRI score of 1-2 will go on to have biopsy, so the final reduction in the number of biopsies was estimated to be 26.4% (33% x 0.8). This real-world scenario assumption is taken from the NICE 2019 update to the prostate cancer diagnosis guidelines, which used expert opinion to derive the 20% figure.[16]

Incidence

By comparison with a biopsy-first approach, MRI followed by biopsy leads to a reduction in the number of clinically *insignificant* cancers (Gleason 6) detected, and an increase in the number of clinically *significant* cancers (Gleason ≥7) detected.[14,15] There have been > 25 studies reporting relevant results. In Drost and colleagues Cochrane systematic review and meta-analysis, the pooled absolute difference in the proportion of men detected with clinically significant and clinically insignificant cancers by MRI and systematic biopsy were:[14]

- 8.2% (95% CI: 6% to 10.3%) reduction in clinically insignificant cancers
- 2% (95% CI: 1.1% to 4.6%) increase in clinically significant cancers

We applied these values to the:

- baseline incidence of prostate cancer, taking into account the age-specific proportion of cancers detected by stage as seen in the ECRIC database of all clinically detected cases in East Anglia, England.[17]
- relative increase in incidence of prostate cancer with screening, adjusting for the age-specific proportion of cancers detected by stage in the ProtecT trial.[17]

When the tumour grade of a prostate cancer is incorrectly assigned, this is known as misclassification. Cancers misclassified as insignificant in a screening programme are likely to go on to become interval cancers, some of which will be clinically detected. Using the results of the Trio study comparing MRI-targeted, systematic, and both MRI-targeted and systematic biopsy, we have estimated the proportion of cancers incorrectly classified as *insignificant* (Gleason 6) as 2.76%, 95% CI: 2.06% to 3.46%.[18]

Mortality

By detecting more clinically significant disease that might otherwise have been detected at a more advanced stage, alongside evidence for the potential of MRI to enhance pre-therapeutic risk assessment to improve outcomes,[19] the use of MRI prior to biopsy is expected to translate into a reduction in mortality. We have adjusted baseline population prostate-cancer–specific mortality, taking into account the age-specific distribution of cancers at diagnosis, to reflect the impact of MRI.

As there are no data to support a change in relative risk of mortality from prostate cancer with screening in the context of MRI, we have not adjusted this relative risk. Our results are therefore likely to be conservative. However, this reduces the number of assumptions where empirical evidence is not available.

Overdiagnosis

Age-specific overdiagnosis was estimated by multiplying the number of cases by (-0.62 + age x 0.014), derived from Pashayan and colleagues estimates of overdiagnosis with PSA screening.[20] To account for the impact of MRI, we then adjusted the proportion of cases estimated to be overdiagnosed with screening by multiplying this figure by the decrease in clinically *insignificant* cancers detected.

We calculated the ratio of overdiagnosed cancers to prostate cancer deaths prevented by dividing overdiagnosed cancers by the change in total number of prostate cancer deaths between no screening and the relevant screening strategy.

eTable 2: Outcomes of biopsy-first no screening and biopsy-first screening for prostate cancer

Screening strategy	Prostate cancer cases (n)	Screen-detected cancers (n)	Overdiag-nosed Cases (n)	Deaths from prostate cancer (n)	MRI (n)	Biopsies (n)	Total life-years (n)	QALYs with prostate cancer (n)	QALYs - healthy (n)	Total QALYs (n)	Total Costs (£)	Net Monetary Benefit (£ 1,000s)
No screening	537,870		-	192,433	333,508	924,050	60,327,337	1,832,919	44,853,750	46,686,669	3,483	930,251
Age-based screening	644,501	307,729	83,006	155,202	399,651	1,926,315	60,454,271	2,757,589	43,940,443	46,698,032	4,386	929,575
Risk-stratified screening (10-year absolute risk)												
2.0%	623,499	274,217	74,308	157,682	386,627	1,801,032	60,445,134	2,640,192	44,061,561	46,701,753	4,295	929,740
2.5%	614,977	257,394	69,898	159,204	381,342	1,741,613	60,439,794	2,585,244	44,117,461	46,702,705	4,233	929,821
3.0%	606,692	240,071	65,335	160,856	376,204	1,681,269	60,434,025	2,529,393	44,174,027	46,703,421	4,172	929,896
3.5%	598,893	222,995	60,815	162,554	371,366	1,622,416	60,428,112	2,474,823	44,229,093	46,703,916	4,115	929,963
4.0%	591,693	206,594	56,457	164,242	366,900	1,566,393	60,422,242	2,422,768	44,281,451	46,704,219	4,062	930,022
4.5%	585,129	191,099	52,323	165,886	362,829	1,513,873	60,416,534	2,373,868	44,330,493	46,704,360	4,013	930,074
5.0%	579,195	176,613	48,446	167,464	359,149	1,465,122	60,411,059	2,328,390	44,375,978	46,704,368	3,969	930,118
5.5%	573,861	163,164	44,835	168,965	355,841	1,420,162	60,405,856	2,286,380	44,417,888	46,704,268	3,929	930,157
6.0%	569,087	150,735	41,489	170,383	352,879	1,378,878	60,400,943	2,247,752	44,456,329	46,704,081	3,893	930,189
6.5%	564,825	139,283	38,397	171,717	350,236	1,341,074	60,396,326	2,212,343	44,491,483	46,703,826	3,860	930,217
7.0%	561,029	128,751	35,547	172,969	347,881	1,306,521	60,392,000	2,179,952	44,523,564	46,703,517	3,830	930,240
7.5%	557,654	119,075	32,922	174,140	345,788	1,274,971	60,387,956	2,150,362	44,552,804	46,703,166	3,804	930,259
8.0%	554,658	110,192	30,507	175,235	343,929	1,246,180	60,384,181	2,123,353	44,579,432	46,702,785	3,780	930,275
8.5%	552,000	102,038	28,286	176,257	342,280	1,219,911	60,380,660	2,098,710	44,603,670	46,702,380	3,759	930,289
9.0%	549,646	94,552	26,243	177,211	340,820	1,195,942	60,377,379	2,076,231	44,625,728	46,701,959	3,740	930,299
9.5%	547,562	87,678	24,364	178,102	339,527	1,174,064	60,374,322	2,055,724	44,645,804	46,701,528	3,723	930,308
10.0%	545,719	81,361	22,634	178,934	338,384	1,154,086	60,371,474	2,037,013	44,664,079	46,701,092	3,707	930,315

In all scenarios shown in eTable 2, both clinically-detected and screen-detected cancers were assumed to follow pre-2019 NICE guidelines for diagnosis (i.e. biopsy-first, then MRI depending on further assessment needs). The number of prostate cancer cases includes the number of overdiagnosed cancers. Net monetary benefit at a willingness-to-pay threshold of £20,000 ($26,000) per QALY gained. Results are the average of 10,000 simulations. To convert to US $, multiply by approximately 1.3. Abbreviations: QALYs, quality-adjusted life-years.

eTable 3: Outcomes of biopsy-first no screening and biopsy-first screening for prostate cancer per 10,000 men

Screening strategy	Prostate cancer cases (n)	Screen-detected cancers (n)	Overdiag -nosed Cases (n)	Deaths from prostate cancer (n)	MRI (n)	Biopsies (n)	Total life-years (n)	QALYs with prostate cancer (n)	QALYs - healthy (n)	Total QALYs (n)	Total Costs (£)	Net Monetary Benefit (£ 1,000s)
No screening	1,200	-	-	429	744	2,061	134,548	4,088	100,037	104,125	7,767,680	2,074,736
Age-based screening	1,437	686	185	346	891	4,296	134,831	6,150	98,000	104,151	9,781,447	2,073,229
Risk-stratified screening (10-year absolute risk)												
2.0%	1,391	612	166	352	862	4,017	134,811	5,888	98,270	104,159	9,579,247	2,073,598
2.5%	1,372	574	156	355	851	3,884	134,799	5,766	98,395	104,161	9,440,576	2,073,779
3.0%	1,353	535	146	359	839	3,750	134,786	5,641	98,521	104,163	9,305,467	2,073,946
3.5%	1,336	497	136	363	828	3,618	134,773	5,520	98,644	104,164	9,177,822	2,074,096
4.0%	1,320	461	126	366	818	3,494	134,760	5,403	98,761	104,164	9,059,413	2,074,227
4.5%	1,305	426	117	370	809	3,376	134,747	5,294	98,870	104,165	8,950,822	2,074,342
5.0%	1,292	394	108	373	801	3,268	134,735	5,193	98,972	104,165	8,851,963	2,074,442
5.5%	1,280	364	100	377	794	3,167	134,723	5,099	99,065	104,164	8,762,400	2,074,527
6.0%	1,269	336	93	380	787	3,075	134,712	5,013	99,151	104,164	8,681,517	2,074,599
6.5%	1,260	311	86	383	781	2,991	134,702	4,934	99,229	104,163	8,608,629	2,074,661
7.0%	1,251	287	79	386	776	2,914	134,692	4,862	99,301	104,163	8,543,037	2,074,713
7.5%	1,244	266	73	388	771	2,844	134,683	4,796	99,366	104,162	8,484,064	2,074,756
8.0%	1,237	246	68	391	767	2,779	134,675	4,736	99,425	104,161	8,431,069	2,074,792
8.5%	1,231	228	63	393	763	2,721	134,667	4,681	99,479	104,160	8,383,463	2,074,821
9.0%	1,226	211	59	395	760	2,667	134,660	4,631	99,529	104,159	8,340,704	2,074,845
9.5%	1,221	196	54	397	757	2,619	134,653	4,585	99,573	104,158	8,302,300	2,074,865
10.0%	1,217	181	50	399	755	2,574	134,646	4,543	99,614	104,157	8,267,809	2,074,880

Results, equivalent to eTable 2, however shown per 10,000 men. In all scenarios shown in eTable 2, both clinically-detected and screen-detected cancers were assumed to follow pre-2019 NICE guidelines for diagnosis (i.e. biopsy-first, then MRI depending on further assessment needs). The number of prostate cancer cases includes the number of overdiagnosed cancers. Net monetary benefit at a willingness-to-pay threshold of £20,000 ($26,000) per QALY gained. Results are the average of 10,000 simulations. To convert to US $, multiply by approximately 1.3. Abbreviations: QALYs, quality-adjusted life-years.

eTable 4: Outcomes of MRI-first no screening and MRI-first screening for prostate cancer per 10,000 men

Screening strategy	Prostate cancer cases (n)	Screen-detected cancers (n)	Overdiag-nosed Cases (n)	Deaths from prostate cancer (n)	MRI (n)	Biopsies (n)	Total life-years (n)	QALYs with prostate cancer (n)	QALYs - healthy (n)	Total QALYs (n)	Total Costs (£)	Net Monetary Benefit (£ 1,000s)
No Screening	1,177	-	-	425	2,022	1,357	134,561	3,987	100,156	104,144	7,741,163	2,075,133
Age-based screening	1,394	636	158	343	4,239	2,845	134,841	5,841	98,344	104,186	9,774,274	2,073,943
Risk-stratified screening (10-year absolute risk)												
2.0%	1,351	567	141	349	3,320	2,228	134,821	5,608	98,584	104,192	9,251,240	2,074,583
2.5%	1,334	533	133	352	3,063	2,056	134,809	5,499	98,694	104,193	9,023,766	2,074,831
3.0%	1,317	497	124	356	2,849	1,912	134,797	5,387	98,806	104,193	8,828,016	2,075,037
3.5%	1,301	462	116	359	2,673	1,795	134,783	5,279	98,915	104,193	8,662,496	2,075,203
4.0%	1,286	428	107	363	2,531	1,699	134,770	5,175	99,018	104,193	8,523,662	2,075,335
4.5%	1,273	396	100	367	2,415	1,621	134,758	5,077	99,116	104,192	8,407,621	2,075,439
5.0%	1,261	366	92	370	2,322	1,559	134,746	4,986	99,206	104,192	8,310,757	2,075,519
5.5%	1,250	338	85	374	2,246	1,508	134,734	4,901	99,289	104,191	8,229,924	2,075,580
6.0%	1,240	313	79	377	2,184	1,466	134,723	4,824	99,366	104,189	8,162,462	2,075,625
6.5%	1,231	289	73	380	2,134	1,433	134,713	4,753	99,435	104,188	8,106,152	2,075,657
7.0%	1,224	267	68	382	2,094	1,406	134,704	4,688	99,499	104,187	8,059,157	2,075,679
7.5%	1,217	247	63	385	2,061	1,384	134,695	4,628	99,558	104,186	8,019,960	2,075,692
8.0%	1,211	229	58	387	2,035	1,366	134,686	4,574	99,611	104,184	7,987,302	2,075,698
8.5%	1,205	212	54	390	2,014	1,352	134,679	4,524	99,659	104,183	7,960,144	2,075,699
9.0%	1,200	197	50	392	1,997	1,340	134,671	4,479	99,703	104,182	7,937,620	2,075,695
9.5%	1,196	182	47	394	1,983	1,331	134,665	4,437	99,743	104,180	7,919,009	2,075,687
10.0%	1,192	169	43	396	1,972	1,324	134,658	4,400	99,779	104,179	7,903,710	2,075,677

In all scenarios shown in eTable 3, both clinically-detected and screen-detected cancers were assumed to have an MRI prior to biopsy. Net monetary benefit at a willingness-to-pay threshold of £20,000 ($26,000) per QALY gained. Based on 10,000 simulations. To convert to US $, multiply by approximately 1.3. Table 3 in the main manuscript is equivalent to eTable 4, but showing results for the full 4.48 million men. Abbreviations: QALYs, quality-adjusted life-years.

eFigure 1: Population age distribution

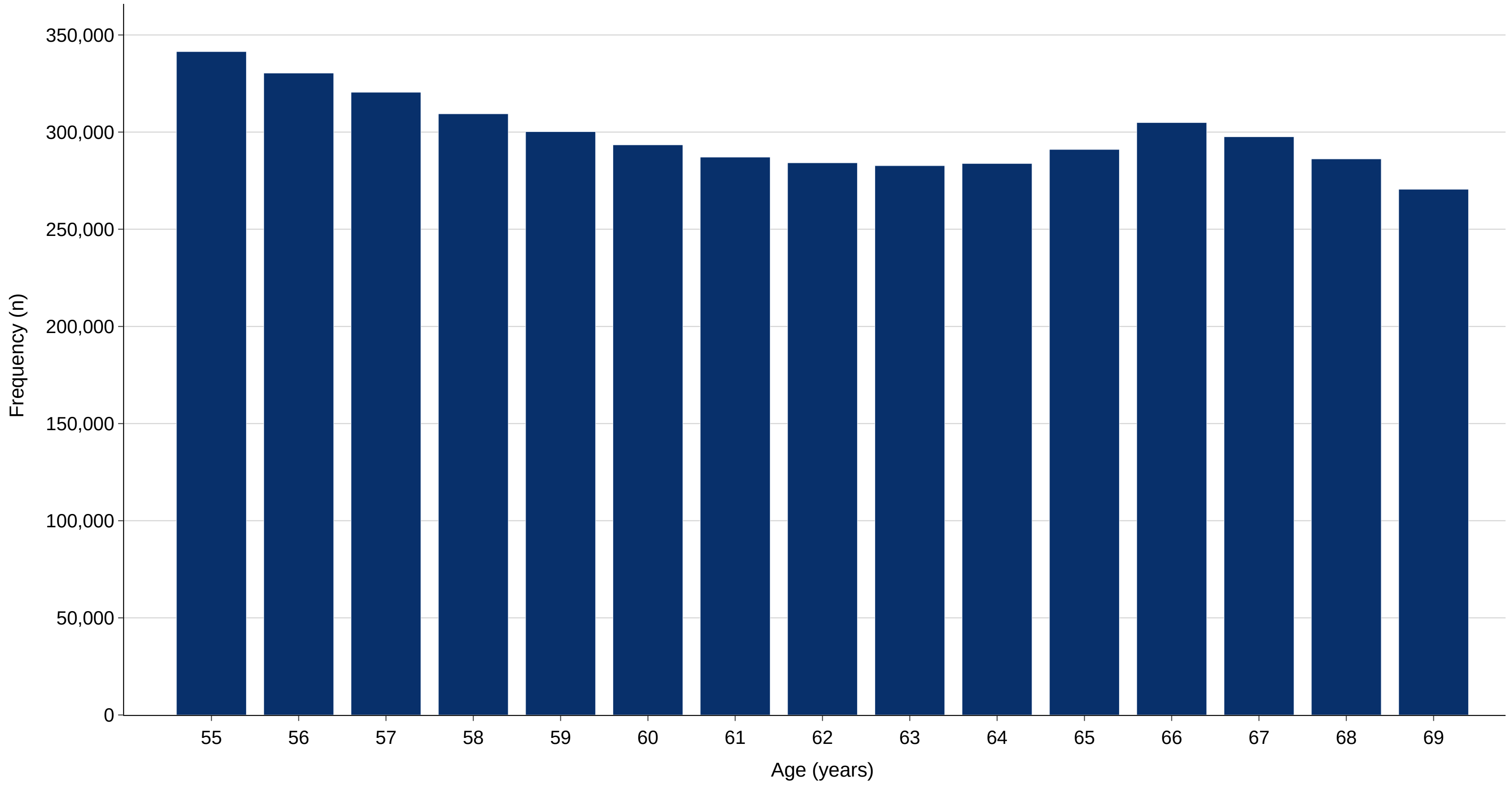

Age distribution of the hypothetical cohorts of 4.48 million men, representing all men in England aged 55-69. Values are an average of the population size in 2013-2016.

© 2021 Callender T et al. *JAMA Network Open*.

eFigure 2: Cost-effectiveness acceptability curves showing MRI-first screening strategies

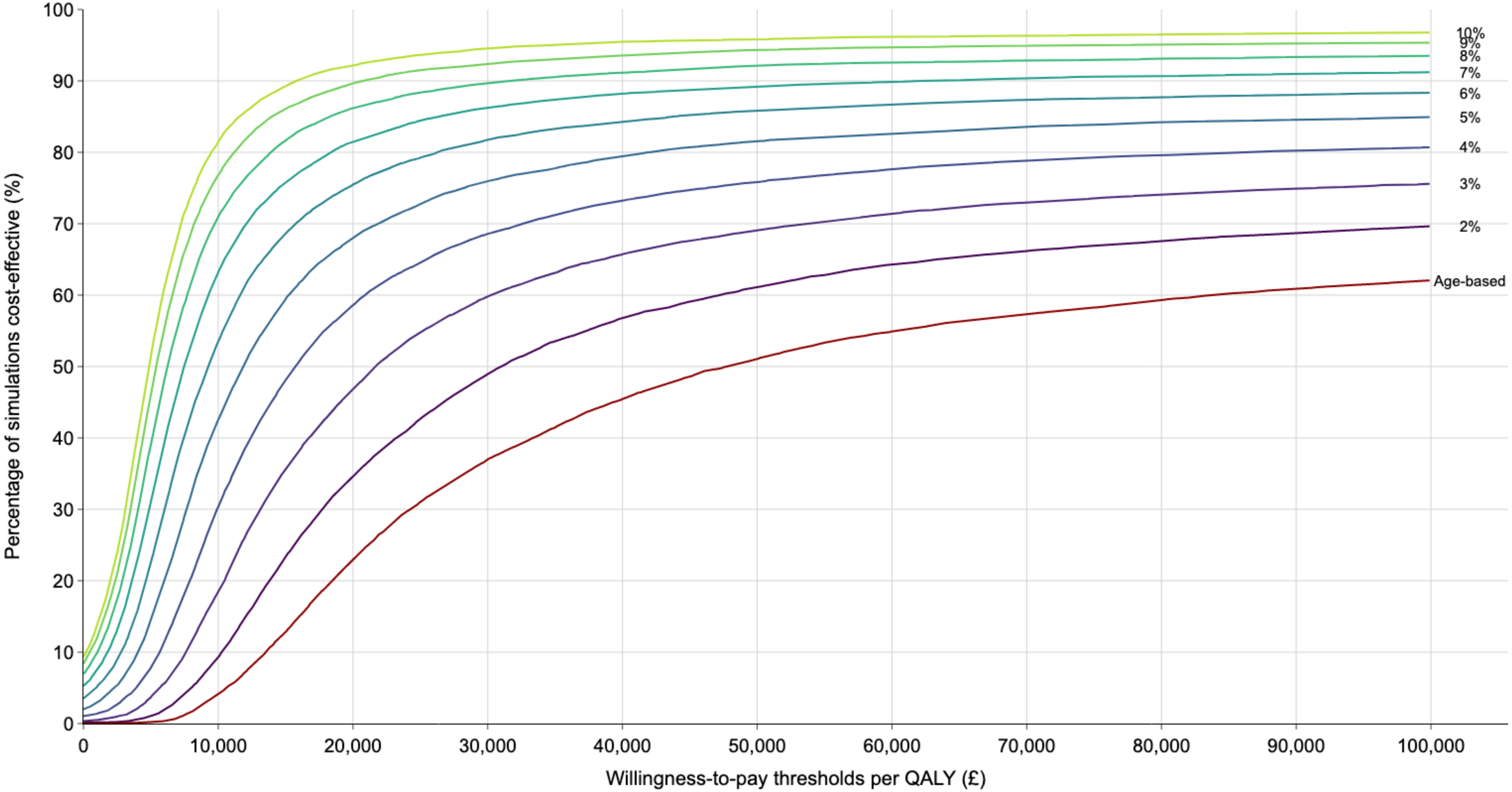

Cost-effectiveness acceptability curves (CEAC) of selected MRI-first screening strategies shown. Each CEAC shows the probability at a specific willingness-to-pay threshold of that strategy having a higher net monetary benefit than no screening. To convert to US $, multiply by approximately 1.3. Abbreviation: QALY, quality-adjusted life-year.

eFigure 3: Cost-effectiveness acceptability frontier showing MRI-first screening strategies

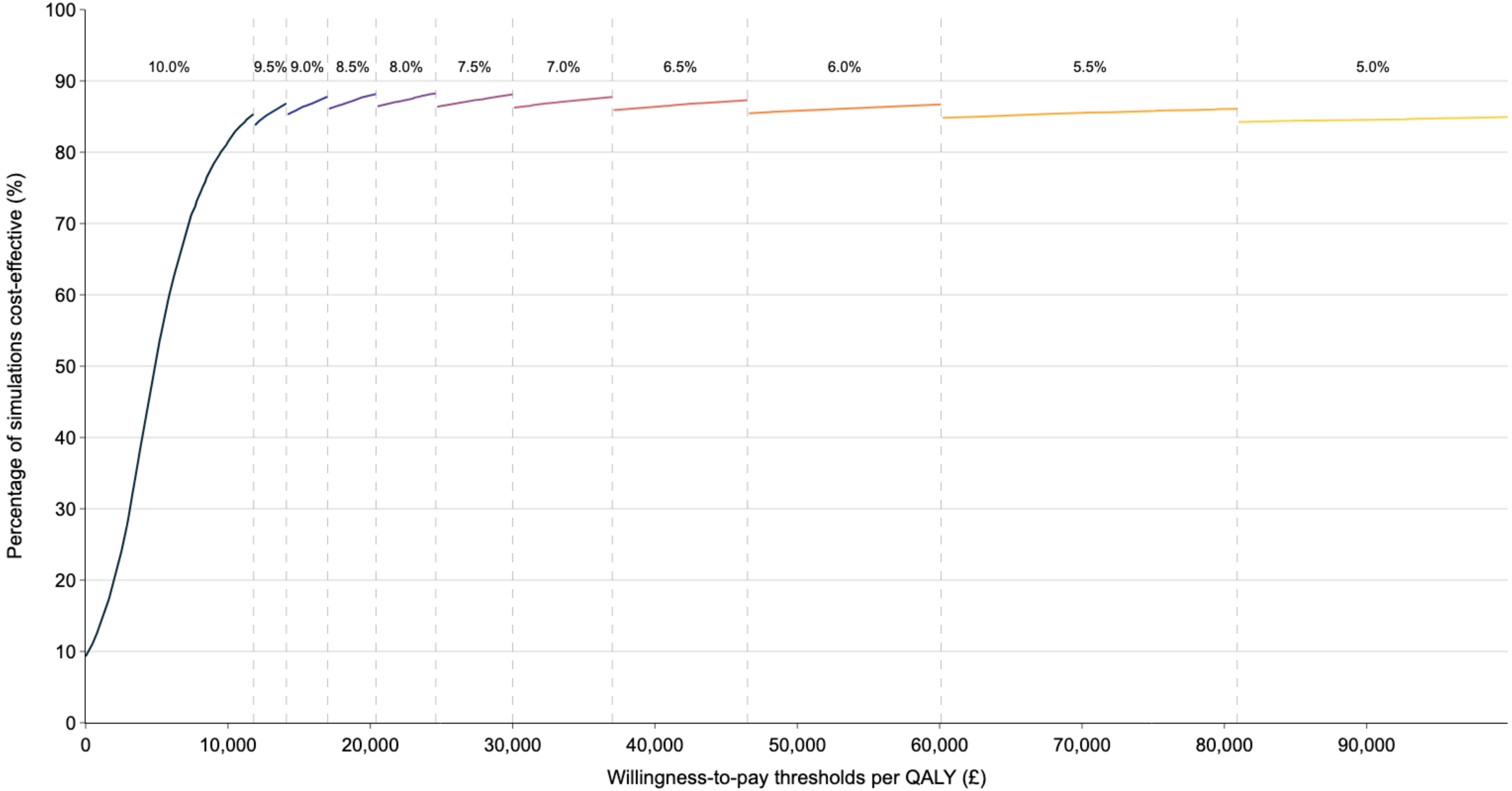

Cost-effectiveness acceptability frontier showing the screening strategy with the highest net monetary benefit at each willingness-to-pay (WTP) threshold, and the probability at that given WTP threshold that the strategy has a higher net monetary benefit than no screening. The strategy is indicated in writing towards the top of the graph (risk-stratified screening at absolute risk thresholds from 10% to 5%). To convert to US $, multiply by approximately 1.3. Abbreviation: QALY, quality-adjusted life-year.

eFigure 4: Net monetary benefit of MRI-first and biopsy-first strategies

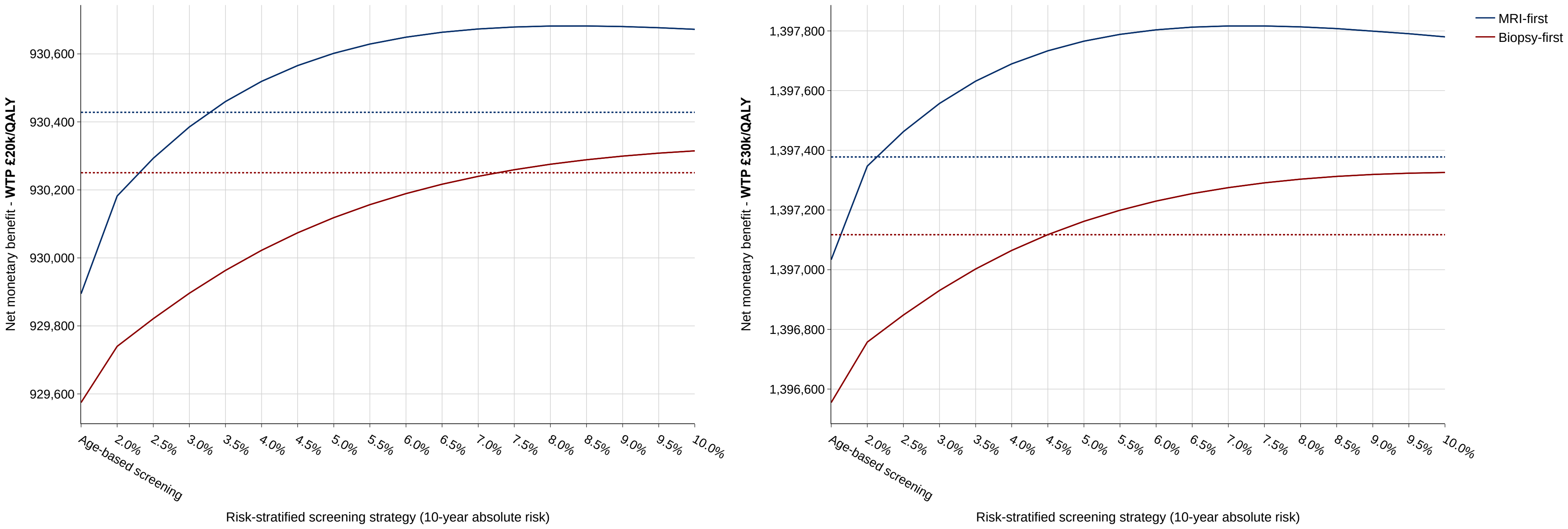

A **B**

Net monetary benefit (NMB) of age-based, risk-stratified, and no screening at willingness-to-pay thresholds of £20,000 (A) and £30,000 (B) per QALY gained. Dotted horizontal lines in blue and red represent the net monetary benefit of no screening. NMB in GBP£ millions; multiply by ~1.3 to convert into US dollars. Abbreviations: NMB, net monetary benefit; WTP, willingness-to-pay; QALY, quality-adjusted life-year.

Sensitivity Analyses

We performed sensitivity analyses to explore the impact of alternative assumptions regarding change in clinically significant and insignificant cancers detected with MRI, as well as different costs of MRI and polygenic risk assessment.

We based our alternative assumptions on the impact of MRI on clinically significant and insignificant cancers on the PRECISION trial.[21] This showed a decrease in the number of clinically *insignificant* cancers detected of -13% (95% CI: -7% to -19%), and an increase in clinically *significant* cancers detected of 12% (95% CI: 4% to 20%). These values were chosen as their confidence intervals include those seen in PROMIS.[13] We re-ran our probabilistic model using these estimates.

eTable 5: Outcomes of no screening and MRI-first screening for prostate cancer per 10,000 men under assumptions from the PRECISION trial

Screening strategy	Prostate cancer cases (n)	Screen-detected cancers (n)	Overdiag-nosed Cases (n)	Deaths from prostate cancer (n)	MRI (n)	Biopsies (n)	Total life-years (n)	QALYs with prostate cancer (n)	QALYs - healthy (n)	Total QALYs (n)	Total Costs (£, 1,000s)	Net Monetary Benefit (£ 1,000s)
No Screening	1,224			403	2,102	1,411	134,635	4,228	99,950	104,178	7,876	2,075,676
Age-based screening	1,445	655	154	325	4,288	2,878	134,902	6,104	98,103	104,207	9,919	2,074,223
Risk-stratified screening (10-year absolute risk)												
2.0%	1,400	585	138	331	3,369	2,262	134,882	5,865	98,349	104,214	9,391	2,074,892
2.5%	1,382	549	130	334	3,114	2,090	134,871	5,754	98,462	104,216	9,161	2,075,154
3.0%	1,364	512	122	337	2,901	1,947	134,859	5,641	98,576	104,217	8,964	2,075,374
3.5%	1,348	476	113	341	2,727	1,830	134,847	5,530	98,688	104,218	8,797	2,075,554
4.0%	1,333	441	105	344	2,585	1,736	134,834	5,424	98,794	104,218	8,657	2,075,701
4.5%	1,319	408	98	348	2,471	1,659	134,822	5,325	98,893	104,218	8,540	2,075,818
5.0%	1,307	377	90	351	2,379	1,597	134,811	5,232	98,986	104,218	8,442	2,075,910
5.5%	1,295	349	84	354	2,304	1,547	134,800	5,147	99,071	104,217	8,361	2,075,983
6.0%	1,285	322	77	357	2,244	1,506	134,789	5,068	99,149	104,217	8,292	2,076,039
6.5%	1,277	298	72	360	2,195	1,473	134,780	4,996	99,220	104,216	8,236	2,076,081
7.0%	1,269	276	66	363	2,156	1,447	134,771	4,930	99,285	104,215	8,188	2,076,112
7.5%	1,262	255	61	365	2,124	1,426	134,762	4,869	99,345	104,214	8,149	2,076,134
8.0%	1,255	236	57	368	2,098	1,409	134,754	4,814	99,399	104,213	8,116	2,076,148
8.5%	1,250	219	53	370	2,078	1,395	134,747	4,764	99,448	104,212	8,088	2,076,156
9.0%	1,245	203	49	372	2,062	1,384	134,740	4,718	99,493	104,211	8,066	2,076,158
9.5%	1,241	188	46	374	2,049	1,376	134,734	4,676	99,534	104,210	8,047	2,076,157
10.0%	1,237	174	42	375	2,039	1,369	134,728	4,638	99,571	104,209	8,031	2,076,152

In all scenarios shown in eTable 4, both clinically-detected and screen-detected cancers were assumed to have an MRI prior to biopsy, under PRECISION trial-based estimates regarding clinically significant and insignificant cancers detected. Net monetary benefit at a willingness-to-pay of £20,000 ($26,000) per QALY gained. Results derived from 10,000 simulations. To convert to US $, multiply by approximately 1.3. Abbreviations: QALYs, quality-adjusted life-years.

eFigure 5: Benefit/harm profile per 10,000 men with MRI-first screening using parameter estimates from the PRECISION trial

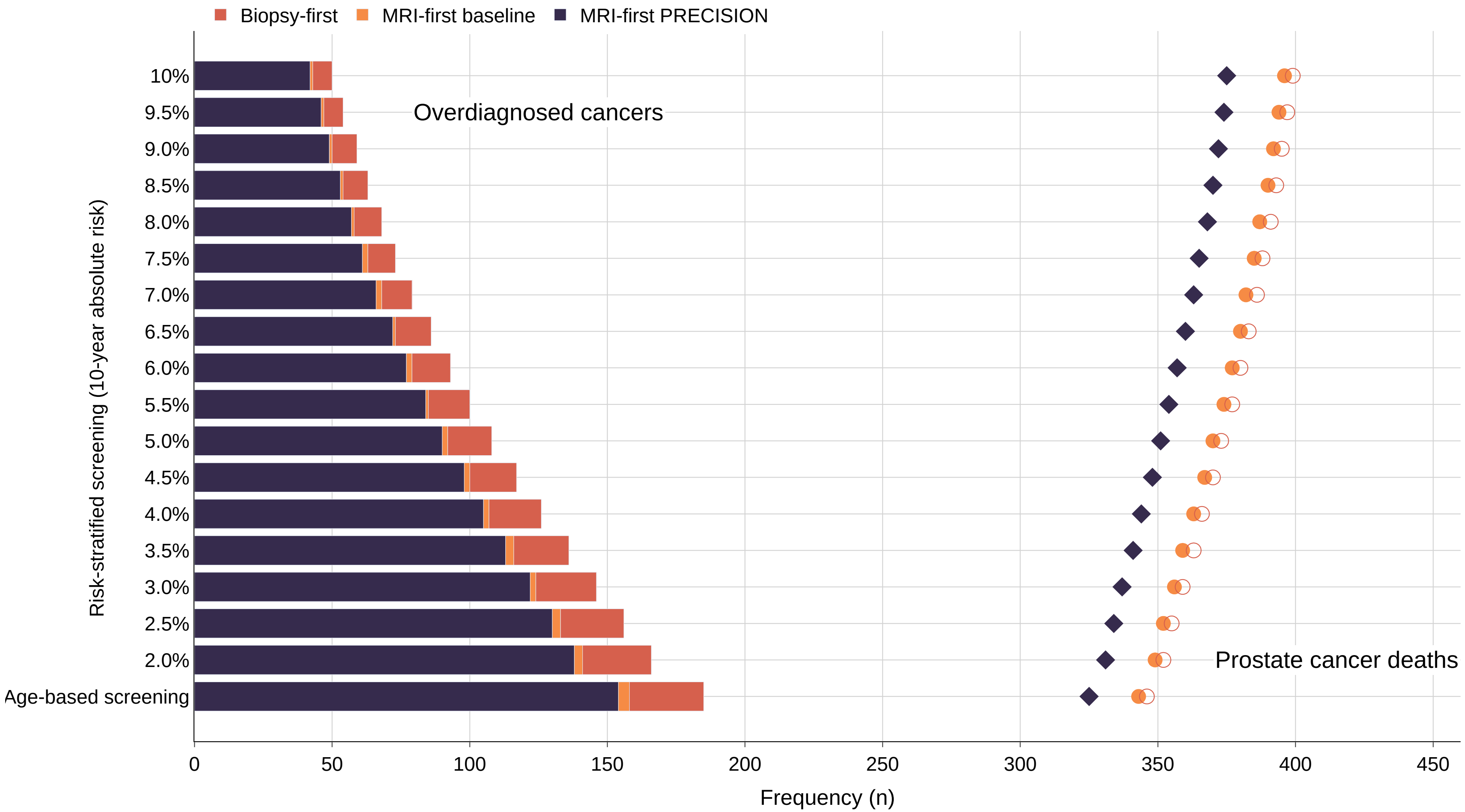

The number of overdiagnosed cancers and prostate cancer-specific deaths per 10,000 men under parameter estimates from PRECISION[21] are shown. The relative changes were:

MRI-first baseline:

- Clinically insignificant cancers: -8.2%, 95% CI: -6 to -10.3
- Clinically significant cancers: 2%, 95% CI: 1.1% to 4.6%

MRI-first PRECISION:

- Clinically insignificant cancers: -13%, 95% CI: -7% to -19%
- Clinically significant cancers: 12%, 95% CI: 4% to 20%

Dark blue diamonds refer to prostate cancer-specific deaths under PRECISION assumptions, yellow filled-in circles under baseline assumptions, and orange open circles with a biopsy-first diagnostic pathway. The bars reflect overdiagnosed cancers.

eFigure 6: Net monetary benefit per 10,000 men of MRI-first screening and no screening using parameter estimates from the PRECISION trial

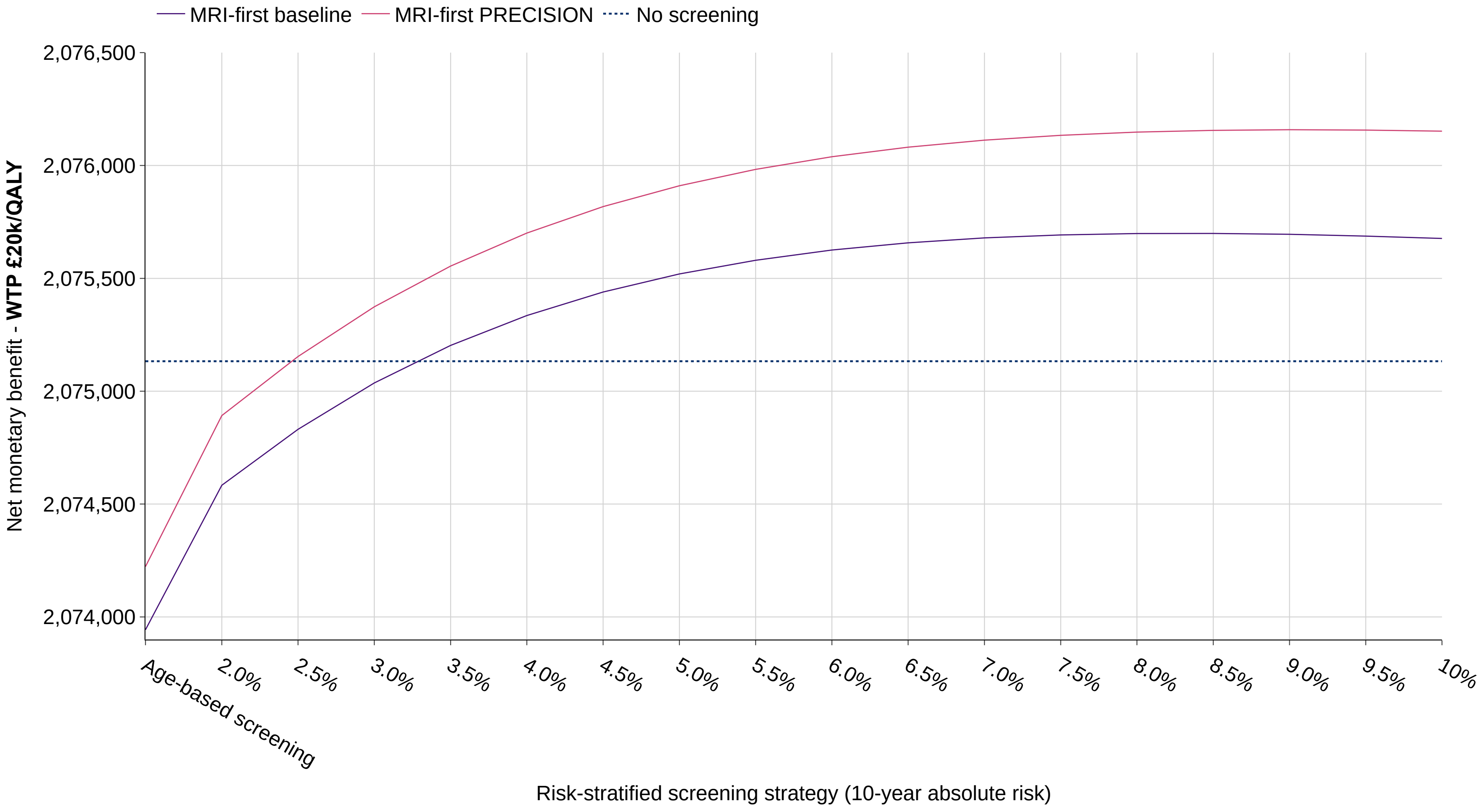

NMB at a WTP threshold of £20,000 ($26,000) per quality-adjusted life-year gained. Results based on 10,000 simulations and displayed in NMB (£1000s). To convert to US $, multiply by approximately 1.3. Abbreviations: NMB, net monetary benefit; WTP, willingness-to-pay; CIS, clinically insignificant cancers; QALY, quality-adjusted life-year.

eFigure 7: Net monetary benefit per 10,000 men of MRI-first screening and no screening at different costs of MRI

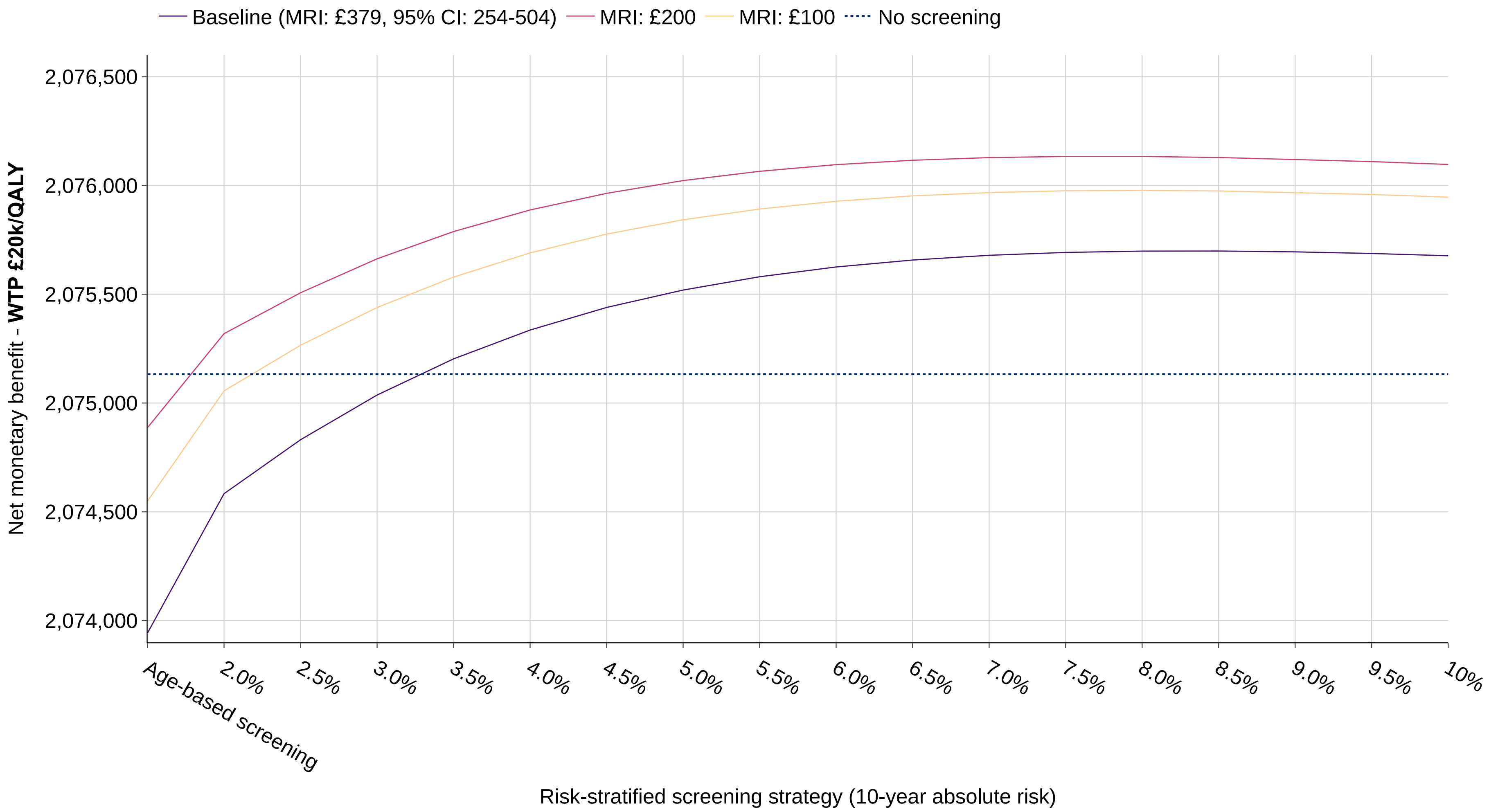

NMB at a WTP of £20,000 ($26,000) per QALY gained. At a cost per MRI of £200 ($260), all MRI-first Risk-stratified screening strategies studied had a higher NMB than no screening. Results based on 10,000 simulations and displayed in NMB (£1000s). To convert to US $, multiply by approximately 1.3. Abbreviations: NMB, net monetary benefit; WTP, willingness-to-pay; CI, confidence intervals; QALY, quality-adjusted life-year.

eFigure 8: Net monetary benefit per 10,000 men of MRI-first screening and no screening at different costs of polygenic risk stratification

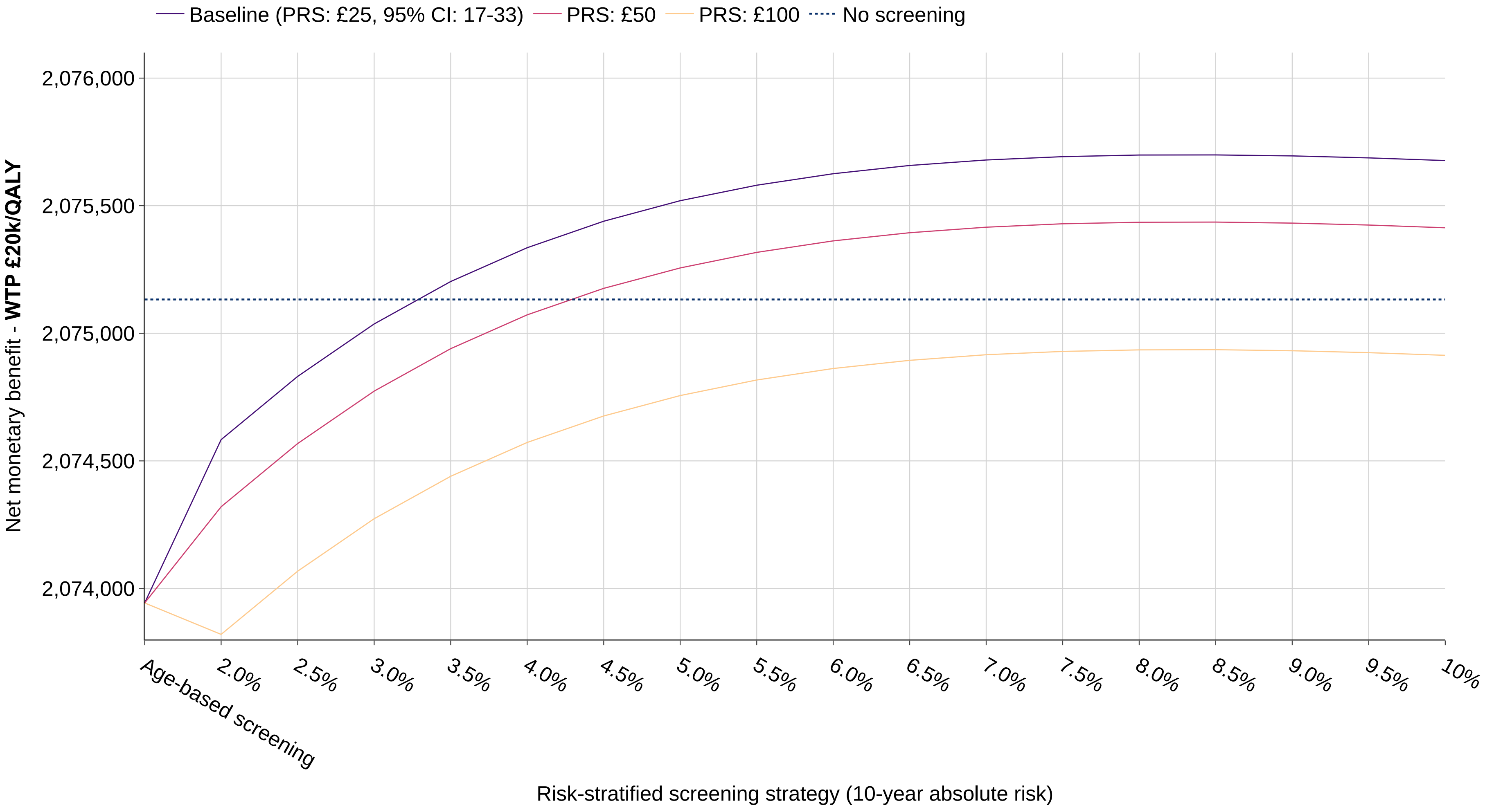

Net monetary benefit at a willingness-to-pay threshold of £20,000 ($26,000) per quality-adjusted life-year gained. Results based on 10,000 simulations and displayed in NMB (£1000s). To convert to US $, multiply by approximately 1.3. Abbreviations: WTP, willingness-to-pay; PRS, polygenic risk score; CI, confidence intervals; QALYs, quality-adjusted life-years.

eFigure 9: Net monetary benefit per 10,000 men of MRI-first screening and no screening at 75% uptake of PSA screening and polygenic risk stratification

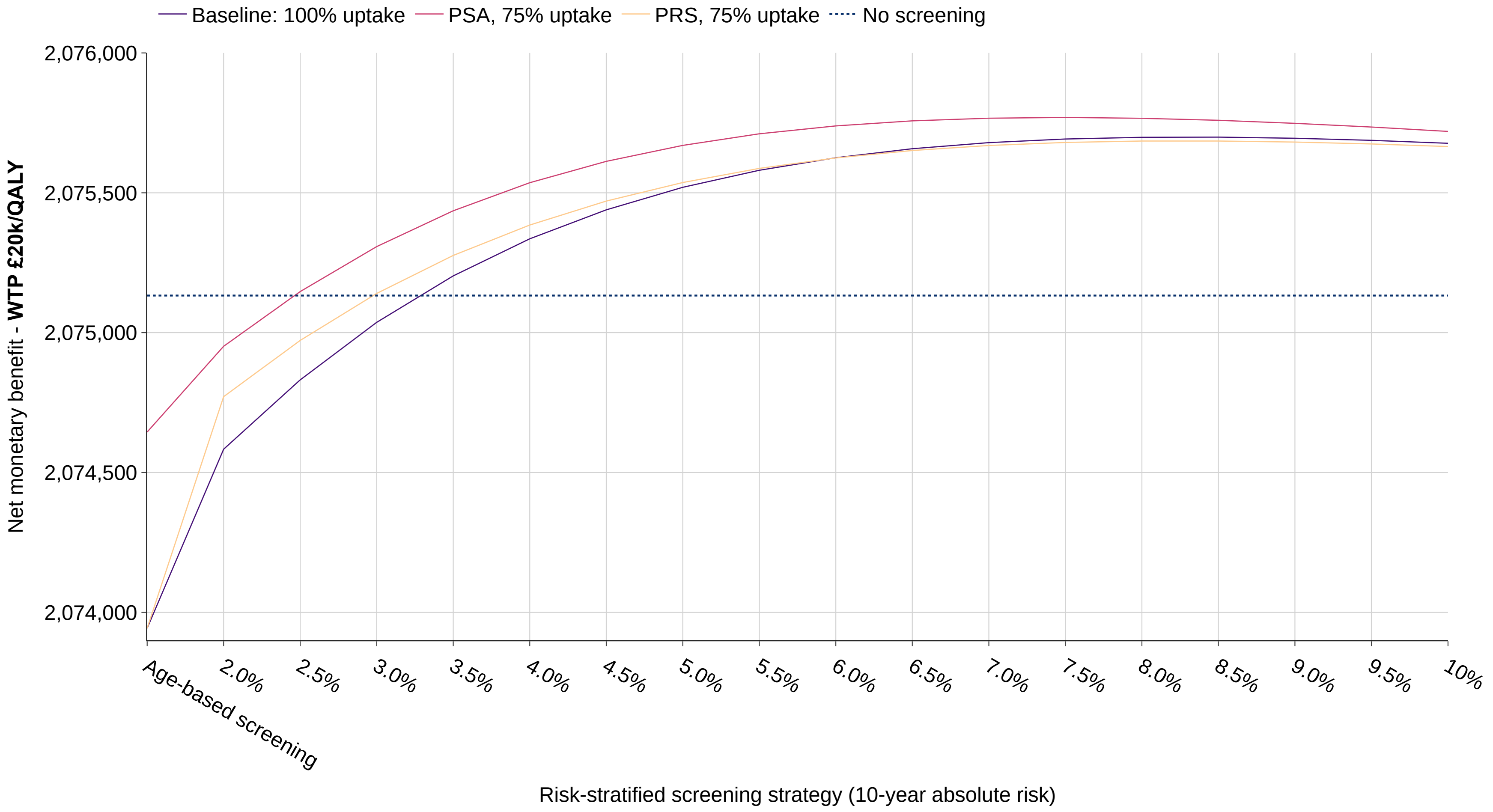

Net monetary benefit (NMB) at a willingness-to-pay threshold of £20,000 ($26,000) per quality-adjusted life-year gained. Each parameter is varied separately. For example, the curve in pink reflects 75% uptake of initial PSA screening, but assumes that of those 75% who take up screening, 100% will undergo risk-stratification in the risk-stratified cohorts. Results based on 10,000 simulations and displayed in NMB (£1000s). To convert to US $, multiply by approximately 1.3. Abbreviations: WTP, willingness-to-pay; PRS, polygenic risk score; CI, confidence intervals; QALYs, quality-adjusted life-years.

eFigure 10: Overdiagnosed cancers and prostate cancer deaths prevented per 10,000 men of MRI-first risk-stratified compared to MRI-first age-based screening when overdiagnosis varies by polygenic risk

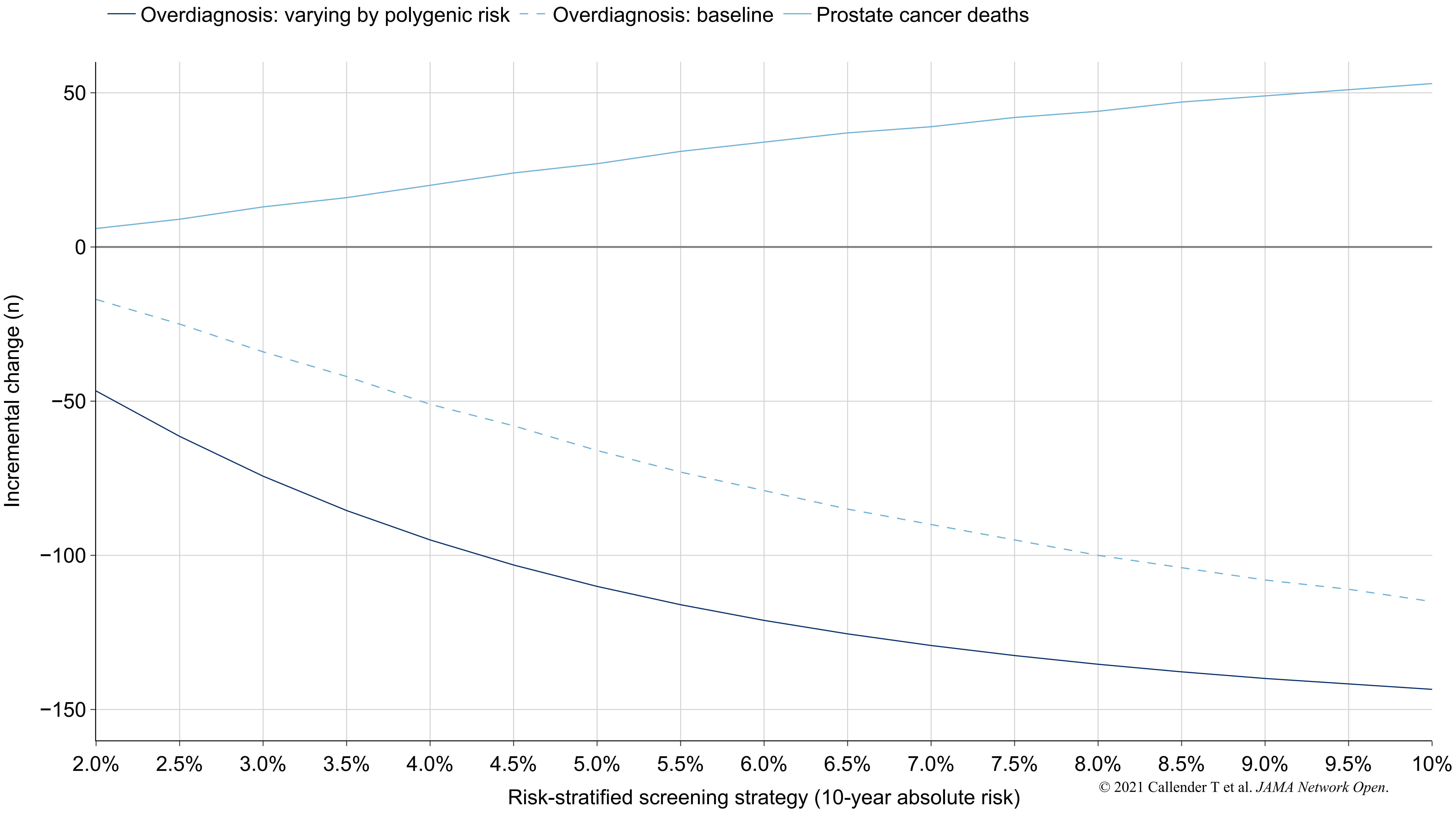

© 2021 Callender T et al. *JAMA Network Open*.

eFigure 11: Observed vs predicted incidence rate

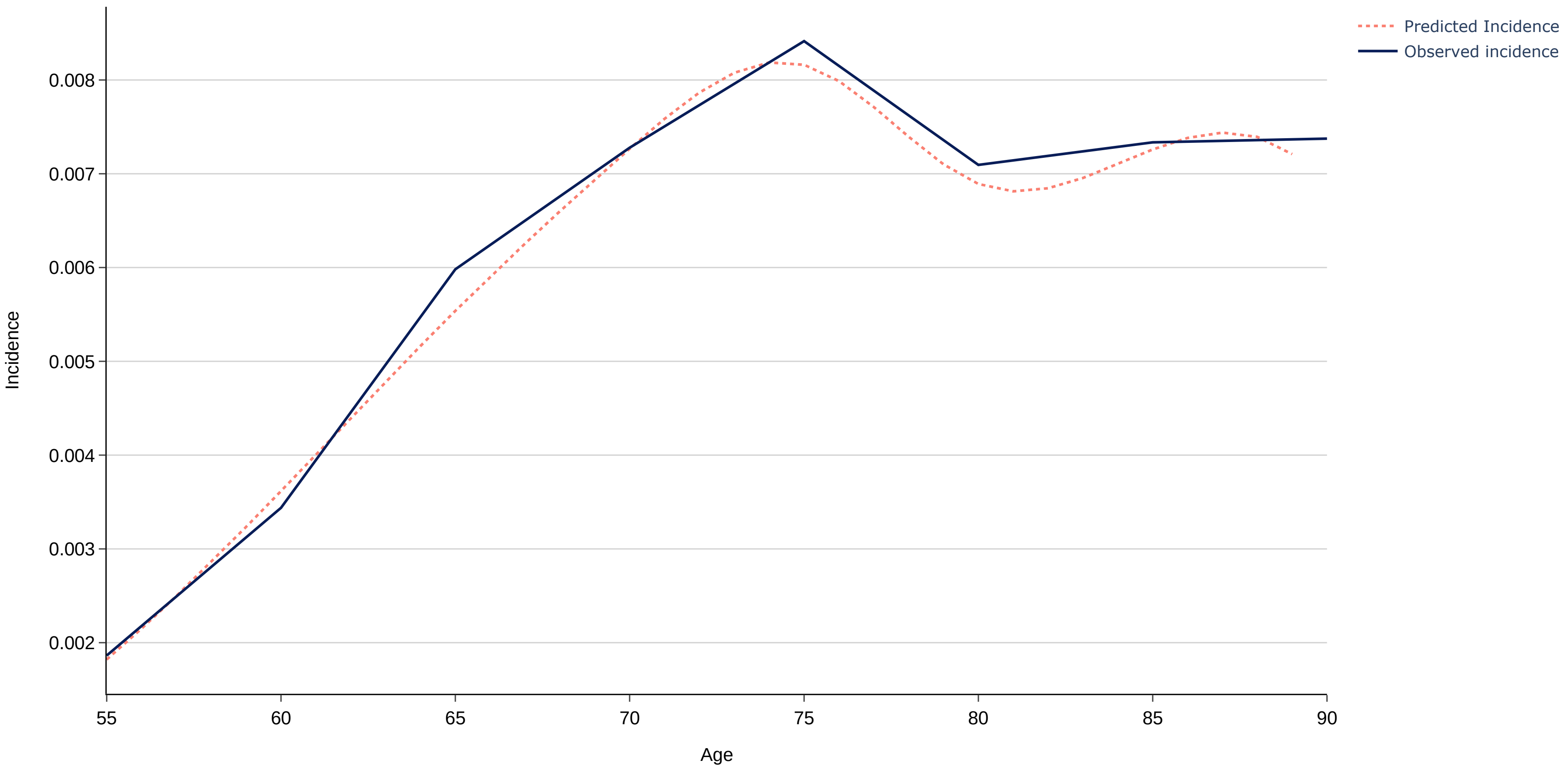

Calibration of predicted incidence rate (in the absence of screening) to the observed rate.

eFigure 12: Observed vs predicted mortality rate

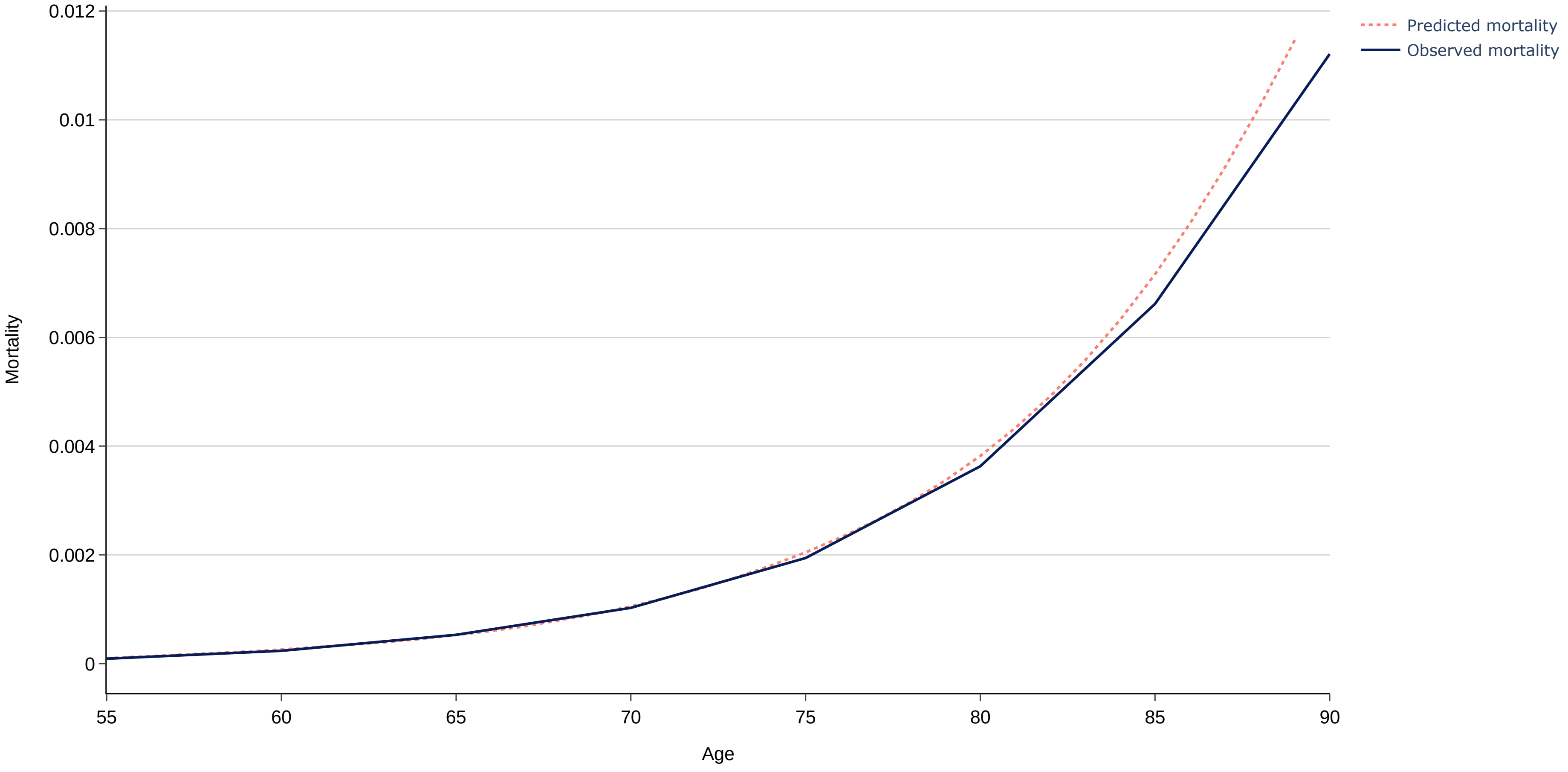

Calibration of predicted mortality rate (in the absence of screening) to the observed rate.

© 2021 Callender T et al. *JAMA Network Open*.

eReferences

1. Callender T, Emberton M, Morris S, et al. Polygenic risk-tailored screening for prostate cancer: A benefit–harm and cost-effectiveness modelling study. *PLOS Med*. 2019;16(12):e1002998. doi:10.1371/journal.pmed.1002998
2. Hjelmborg JB, Scheike T, Holst K, et al. The Heritability of Prostate Cancer in the Nordic Twin Study of Cancer. *Cancer Epidemiol Biomarkers Prev*. 2014;23(11):2303-2310. doi:10.1158/1055-9965.EPI-13-0568
3. Mucci LA, Hjelmborg JB, Harris JR, et al. Familial Risk and Heritability of Cancer Among Twins in Nordic Countries. *JAMA*. 2016;315(1):68. doi:10.1001/jama.2015.17703
4. Pharoah PDP, Antoniou A, Bobrow M, Zimmern RL, Easton DF, Ponder BAJ. Polygenic susceptibility to breast cancer and implications for prevention. *Nat Genet*. 2002;31(1):33-36. doi:10.1038/ng853
5. Dadaev T, Saunders EJ, Newcombe PJ, et al. Fine-mapping of prostate cancer susceptibility loci in a large meta-analysis identifies candidate causal variants. *Nat Commun*. 2018;9(1):2256. doi:10.1038/s41467-018-04109-8
6. NICE. *NICE Guideline NG131: Prostate Cancer: Biology, Diagnosis and Management*. London, U.K.; 2019.
7. National Collaborating Centre for Cancer. *Prostate Cancer: Diagnosis and Treatment: Clinical Guideline*. London; 2014. https://www.nice.org.uk/guidance/CG175.
8. Rosario DJ, Lane JA, Metcalfe C, et al. Short term outcomes of prostate biopsy in men tested for cancer by prostate specific antigen: prospective evaluation within ProtecT study. *BMJ*. 2012;344(jan09 1):d7894-d7894. doi:10.1136/bmj.d7894
9. Department of Health. NHS reference costs 2015 to 2016. https://www.gov.uk/government/publications/nhs-reference-costs-2015-to-2016. Accessed July 19, 2018.
10. Office for National Statistics. CPI Index. 2020. https://www.ons.gov.uk/economy/inflationandpriceindices/timeseries/d7fc/mm23. Accessed March 20, 2020.
11. Hamdy FC, Donovan JL, Lane JA, et al. 10-Year Outcomes after Monitoring, Surgery, or Radiotherapy for Localized Prostate Cancer. *N Engl J Med*. 2016;375(15):1415-1424. doi:10.1056/NEJMoa1606220
12. Leal J, Welton NJ, Martin RM, et al. Estimating the sensitivity of a prostate cancer screening programme for different PSA cut-off levels: A UK case study. *Cancer Epidemiol*. 2018;52:99-105. doi:10.1016/j.canep.2017.12.002
13. Ahmed HU, El-Shater Bosaily A, Brown LC, et al. Diagnostic accuracy of multi-parametric MRI and TRUS biopsy in prostate cancer (PROMIS): a paired validating confirmatory study. *Lancet*. 2017;389(10071):815-822. doi:10.1016/S0140-6736(16)32401-1
14. Drost F-JH, Osses DF, Nieboer D, et al. Prostate MRI, with or without MRI-targeted biopsy, and systematic biopsy for detecting prostate cancer. *Cochrane Database Syst Rev*. 2019;18(1):e728-e729. doi:10.1002/14651858.CD012663.pub2
15. Elwenspoek MMC, Sheppard AL, McInnes MDF, et al. Comparison of Multiparametric Magnetic Resonance Imaging and Targeted Biopsy With Systematic Biopsy Alone for the Diagnosis of Prostate Cancer. *JAMA Netw Open*. 2019;2(8):e198427. doi:10.1001/jamanetworkopen.2019.8427
16. NICE. *Resource Impact Report: Prostate Cancer: Diagnosis and Management (Update) (NG131)*.; 2019. https://www.nice.org.uk/guidance/ng136/resources/resource-impact-report-pdf-6899957101.
17. Pashayan N, Pharoah P, Neal DE, et al. Stage shift in PSA-detected prostate cancers - Effect modification by Gleason score. *J Med Screen*. 2009;16(2):98-101. doi:10.1258/jms.2009.009037
18. Ahdoot M, Wilbur AR, Reese SE, et al. MRI-Targeted, Systematic, and Combined Biopsy for Prostate Cancer Diagnosis. *N Engl J Med*. 2020;382(10):917-928. doi:10.1056/NEJMoa1910038
19. Dell'Oglio P, Stabile A, Dias BH, et al. Impact of multiparametric MRI and MRI-targeted biopsy on pre-therapeutic risk assessment in prostate cancer patients candidate for radical prostatectomy. *World J Urol*. 2019;37(2):221-234. doi:10.1007/s00345-018-2360-1
20. Pashayan N, Duffy SW, Pharoah P, et al. Mean sojourn time, overdiagnosis, and reduction in advanced stage prostate cancer due to screening with PSA: Implications of sojourn time on screening. *Br J Cancer*. 2009;100(7):1198-1204. doi:10.1038/sj.bjc.6604973
21. Kasivisvanathan V, Rannikko AS, Borghi M, et al. MRI-Targeted or Standard Biopsy for Prostate-Cancer Diagnosis. *N Engl J Med*. 2018;378(19):1767-1777. doi:10.1056/NEJMoa1801993
22. Pashayan N, Duffy SW, Neal DE, et al. Implications of polygenic risk-stratified screening for prostate cancer on overdiagnosis. *Genet Med*. 2015;17(10):789-795. doi:10.1038/gim.2014.192
